# Himalayan Aromatic Medicinal Plants: A Review of their Ethnopharmacology, Volatile Phytochemistry, and Biological Activities

**DOI:** 10.3390/medicines3010006

**Published:** 2016-02-19

**Authors:** Rakesh K. Joshi, Prabodh Satyal, Wiliam N. Setzer

**Affiliations:** 1 Department of Education, Government of Uttrakhand, Nainital 263001, India; raakeshjoshi@rediffmail.com; 2 Department of Chemistry, University of Alabama in Huntsville, Huntsville, AL 35899, USA; prabodhsatyal@gmail.com

**Keywords:** Jammu and Kashmir, Himachal Pradesh, Uttarakhand, Nepal, Sikkim, Bhutan, essential oils

## Abstract

Aromatic plants have played key roles in the lives of tribal peoples living in the Himalaya by providing products for both food and medicine. This review presents a summary of aromatic medicinal plants from the Indian Himalaya, Nepal, and Bhutan, focusing on plant species for which volatile compositions have been described. The review summarizes 116 aromatic plant species distributed over 26 families.

## 1. Introduction

The Himalya Center of Plant Diversity [1] is a narrow band of biodiversity lying on the southern margin of the Himalayas, the world’s highest mountain range with elevations exceeding 8000 m. The plant diversity of this region is defined by the monsoonal rains, up to 10,000 mm rainfall, concentrated in the summer, altitudinal zonation, consisting of tropical lowland rainforests, 100–1200 m asl, up to alpine meadows, 4800–5500 m asl. Hara and co-workers have estimated there to be around 6000 species of higher plants in Nepal, including 303 species endemic to Nepal and 1957 species restricted to the Himalayan range [2,3,4]. The Indian Himalaya is home to more than 8000 species of vascular plants [5] of which 1748 are known for their medicinal properties [6].

**Figure 1:**
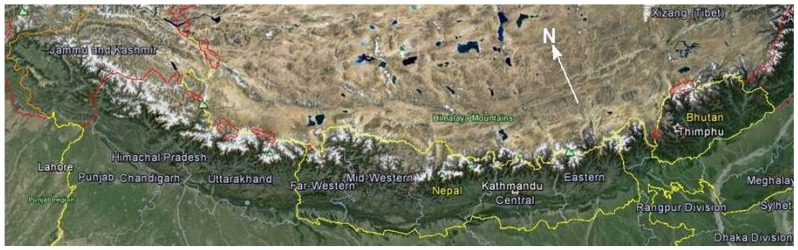
Google Earth^©^ map of the Himalayan region.

Higher plants have played key roles in the lives of tribal peoples living in the Himalaya by providing forest products for both food and medicine. Numerous wild and cultivated plants have been utilized as curative agents since ancient times, and medicinal plants have gained importance recently, not only as herbal medicines, but also as natural ingredients for the cosmetic industry. In this review, we summarize aromatic medicinal plants from Bhutan, Nepal, and the Indian Himalaya of Uttarakhand, Himachal Pradesh, and Jammu and Kashmir (Figure 1). We have focused the review on plant species for which volatile compositions have been described. In searching the literature (Google Scholar), we have used the keywords: essential oil, Himalaya, Bhutan, Nepal, Uttarakhand, Himachal Pradesh, and Kashmir. For essential oils from these regions that were reported in the literature, we have carried out an additional search using the plant name and the keywords, ethnobotany, ethnopharmacology.

Table 1 summarizes the aromatic medicinal plants of the Himalayan region and includes ethnopharmacological uses of the plants, essential oil compositions, and any biological activities of the essential oils. In addition, we describe in more detail some important genera and species used as aromatic medicinal plants in this region.

## 2. The Genus *Artemisia*

There are approximately 400 species of *Artemisia* distributed throughout temperate regions of the world, and the genus is typically characterized by aromatic shrubs and herbs [299]. Numerous members of the genus are used as traditional medicines by indigenous cultures, and many show biological activities including antimalarial, cytotoxic, antihepatotoxic, antibacterial, antifungal and antioxidant activities [300]. Some particularly notable members of the genus include *A. absinthium* L., the major component of the notorious spirit drink absinthe [301]; *A. annua* L., the efficacious antimalarial drug qinghaosu [302]; *A. dracunculus* L., the flavoring herb tarragon [303]; and *A. tridentata* Nutt., the “big sagebrush” of western North America [304].

In the Himalaya, 19 species of *Artemisia* are recognized to be medicinal herbs (*A. absinthium*, *A. biennis*, *A. brevifolia*, *A. desertorum*, *A. dracunculus*, *A. dubia*, *A. gmelinii*, *A. indica*, *A. japonica*, *A. lacinata*, *A. macrocephala*, *A. maratima*, *A. moorcroftiana*, *A. nilagarica*, *A. parviflora*, *A. roxburghiana*, *A. scoparia*, *A. sieversiana*, and *A. vulgaris*) [55,59], and some of these have been investigated for volatile compositions and bioactivity (see Table 1). *A. dracunculus* (tarragon) is used worldwide, including the Himalayan region, as a flavoring agent for food. The plant is also used ethnobotanically. Native peoples in the Nubra valley (Kashmir) [38], Kibber Wildlife Sanctuary (Himachal Pradesh) [39], and the Lahaul Valley (Himachal Pradesh) [40] use a paste from the leaves to treat wounds on the legs of donkeys and yaks; an extract of the whole plant is used to relieve toothache, reduce fever, and as a treatment for dysentery, intestinal worms, and stomachache. *A. dracunculus* from the Himalayas is a rich source of the diacetylene capillene and the monoterpene (*Z*)-β-ocimene [36,37,41], and is markedly different from “French tarragon”, which is dominated by estragole (up to 74%), or “Russian tarragon”, which is dominated by elemicin (up to 57%), or other cultivars of *A. dracunculus* [303].

The leaf juice of *A. dubia* is used by villagers in the Dolpa district of Nepal [285] and the Newar community of Kathmandu, Nepal [42], as an antiseptic to cure cuts and wounds and the leaf extracts are used as pesticides. The essential oil of *A. dubia* was shown to be rich in chrysanthenone (29.0%), coumarins (18.3%), and camphor (16.4%) [43]. Although the leaf oil showed *in vitro* cytotoxic activity against MCF-7 human breast tumor cells and antifungal activity against *Aspergillus niger*, it was inactive against the bacteria *Bacillus cereus*, *Staphylococcus aureus*, *Escherichia coli*, and *Pseudomonas aeruginosa* [43]. Thus, the antiseptic qualities of *A. dubia* must be due to non-volatile components in the plant.

In the Humla district of northwestern Nepal, the whole fresh plant of *A. gmelinii* is ground into a paste an applied externally to cure headache, boils, and pimples [44]. The essential oils from the aerial parts of *A. gmelinii* from Himalayan India are dominated by artemisia ketone and 1,8-cineole [45,46]. Neither of these compounds, however, are notably antibacterial (*B. cereus*, *S. aureus*, *E. coli*, *P. aeruginosa*) or antifungal (*Candida albicans*, *A. niger*) [305].

The essential oil composition of *A. indica* has shown wide variation. The leaf essential oil from Nepal was dominated by ascaridole (15.4%), isoascaridole (9.9%), *trans-p*-mentha-2,8-dien-1-ol (9.7%), and *trans*-verbenol (8.4%) [43]. Conversely, the essential oil from the aerial parts of a sample from Uttarakhand, India was rich in davanone (30.8%), β-pinene (15.3%), and germacrene D (5.8%) [48], while the aerial parts essential oil from a sample collected from Kashmir was dominated by artemisia ketone (42.1%), germacrene B (8.6%), and borneol (6.1%) [47]. The oil from Kashmir was screened for antimicrobial activity and showed extraordinary activity against *S. aureus* and *Penicillium chrysogenum* (MIC = 16 μg/mL). The Kashmir oil also showed remarkable cytotoxic activity against THP-1 (leukemia), A-549 (lung), HEP-2 (liver) and Caco-2 (colon) human tumor cells. The Nepali *A. indica* oil showed neither antibacterial (*B. cereus*, *S. aureus*, *E. coli*, *P. aeruginosa*), antifungal (*A. niger*), nor cytotoxic (MCF-7 breast tumor) activities [43]. In Nepal, the leaves are used to make a paste that is applied to cuts and wounds [11,12], while the juice of the plant is used to treat indigestion [42].

In the Garhwal Himalaya, Uttarakhand, the leaves of *Artemisia japonica* are used as an incense and insecticide [49] and in ethnoveterinary medicine the plant is used as a treatment for internal parasites (e.g., round worm) [306]. In northern Pakistan, the leaf extract is used to treat malaria while a paste of the leaves is applied externally to treat skin diseases [50]. The essential oil from the aerial parts of *A. japonica* collected from Milam glacier (Uttarakhand), India, was dominated by the monoterpenoids linalool (27.5%), (*E*)-β-ocimene (6.5%), 1,8-cineole (5.5%), and (*Z*)-β-ocimene (5.5%), along with germacrene D (11.2%) [51]. In contrast, a sample of *A. japonica* from southern India (Munmar, Kerala) was rich in sesquiterpene hydrocarbons: Spathulenol (12%), germacrene D (7.5%), β-elemene (2.8%), β-caryophyllene (2.4%) [307].

*Artemisia maritima* is used by several Himalayan peoples to treat stomach problems and for expelling intestinal worms [50,182,308]. Mathela and co-workers [45] found *A. maritima* essential oil from Malari (Garhwal region, India) to be rich in α-thujone (63.3%), sabinene (7.8%), and 1,8-cineole (6.5%), while 1,8-cineole and chrystanthenone dominated the essential oils from Himachal Pradesh [50] and Chamoli (Garhwal region, India) [51]. Camphor was the dominant monoterpenoid (44.4%) in an essential oil sample from Lahaul-Spiti (Hamachal Pradesh, India) [52], which was screened for antimicrobial activity (*S. aureus*, *E. coli*, *S. abony*, *P. aeruginosa*, *C. albicans*), but was found to be inactive. Commercial *A. maritima* oil from Pakistan was also rich in 1,8-cineole (41.1%) and camphor (20.3%) [309]. α-Thujone has shown anthelmintic activity [310], and high concentrations of α-thujone in some *A. maritima* essential oils likely account for the ethnopharmacological use of this plant to expel intestinal parasites. The compound is a potent neurotoxin and modulator of the GABA-gated chloride channel, however [311]. Conversely, camphor has been shown not have anthelmintic activity [312], but the compound is toxic to humans and ingestion may cause seizures [313,314]. 1,8-Cineole has been shown to inhibit castor oil-induced diarrhea in rats [315], prevent ethanol-induced gastric injury in rats [316], and attenuate trinitrobenzene sulfonic acid-induced colitis in rats [317], and so this compound may be an important component in the traditional use of 1,8-cineole-containing herbal medicines for stomach problems.

*Artemisia nilagirica* is widely found in the hilly areas of northern India, where it is used as an insecticide [318]. *A. nilagirica* essential oil compositions have shown altitudinal variation. Badoni and co-workers [55] found that *A. nilagririca* from lower altitudes in Uttarakhand (500 m asl) contained α-thujone (36.9%) as the major component, the oil from intermediate elevation (1200 m asl) had mequinyl *p*-nitrobenzoate (22.1%), cadina-1,4-diene (17.7%), and β-eudesmol (12.4%) as the major components, and the sample from higher elevation (2000 m asl) had linalool (32.5%) and isopulegyl acetate (20.7%) as the major components. Haider and co-workers [56], working in Himachal Pradesh, observed a similar effect, albeit with very different composition. *A. nilagririca* from lower altitudes (Mandi, 1044 m asl) contained caryophyllene oxide (28.6%) as the major component, the oil from intermediate elevation (Manali, 2050 m asl) had borneol (35.8%) as the major component, and the sample from higher elevation (Shimla, 2210 m asl) was dominated by camphor (46.9%).

The *A. nilagirica* essential oil from Himachal Pradesh [major components: camphor (12.6%), artemisia ketone (10.2%), caryophyllene oxide (7.4%), borneol (5.3%)] showed antifungal activity against the plant pathogenic fungi *Colletotrichum acutatum*, *Colletotrichum fragariae*, and *Colletotrichum gloeosporioides*, but did not show antimicrobial activity against *S. aureus*, *E. coli*, *S. abony*, *P. aeruginosa*, or *C. albicans* [52]. Similarly, the α-thujone-rich essential oil from Uttarakhand was active against plant pathogenic fungi *Rhizoctonia solani*, *Sclerotium rolfsii*, and *Macrophomina phaseolina* [54]. Another essential oil sample from Uttarakhand [major components: linalool (16.3%), α-thujone (13.9%), β-caryophyllene (7.5%), germacrene D (7.1%)] did show notable antibacterial activity against *S. aureus* and *P. aeruginosa* with MIC values of 6.25 and 12.5 μg/mL, respectively [55]. Traditional medical practitioners in Darjeeling, West Bengal, India, chew shoots of the plant to treat oral ulcers and apply crushed leaves to the forehead for dizziness and headaches [54]. Inhabitants of the Parvati valley, Himachal Pradesh, India, make a paste from the leaves and apply it cuts and wounds to check bleeding [53]. The antimicrobial activities of *A. nilagrica* (see above) are consistent with the traditional uses of the plant for wounds and ulcers.

*Artemisia parviflora* is widely distributed in the Himalayas between about 900 and 3500 m asl [319]. In the traditional medicine of the Kumaun Himalaya, the leaves of *A. parviflora* are used to treat skin diseases, burns, cuts, and wounds, while the volatiles from the plant are used to repel insects [19]. The indigenous peoples of Jammu and Kashmir (India) use *A. parviflora* as a diuretic and to treat gynecological disorders [59]. The plant is also used in ethnoveterinary medicine as an anthelmintic; a decoction of the leaves and buds of the plant are given to stock animals (e.g., horses, mules, sheep, and buffaloes) for round worm [320]. The plant is also used as a fodder plant in mid-altitude rangelands of Uttarakhand [321]. The essential oil from the aerial parts of *A. parviflora* collected from Pauri, Pauri Garhwal (Uttarakhand, India) was found to contain β-caryophyllene (15.3%), germacrene D (14.7%), camphor (11.4%), artemisia ketone (7.8%), and 1,8-cineole (5.8%) [61]. There are apparently no reports on the bioactivities of Himalayan *A. parviflora* essential oil, but the oil from southern India has shown antifungal activity against *Candida* and *Cryptococcus* species [322].

People living in the Kedarnath Wildlife Sanctuary in the western Himalaya of Chamoli-Rudraprayag (Uttarakhand), India, use an extract of the whole plant to relieve fever [49]. In addition, the plant extract is rubbed on the skin to treat allergic reactions. In Jammu and Kashimir, India, *A. roxburghiana* is also used to treat skin allergies [62]. In northern Pakistan, an extract of the whole *A. roxburghiana* plant is used to treat fever and malaria; a powder of the whole plant is taken for intestinal worms [50]. Indigenous people living in the Khyber Pakhtunkhwa Province of Pakistan use the leaves of *A. roxburghiana* to treat chest cold, sore throat, and toothache [323]. *A. roxburghiana* is used in ethnoveterinary medicine in Uttarakhand, India, to treat eye diseases, wounds, cuts, and external parasites [306].

As seen with other *Artemisia* species, there is a wide variation in the essential oil compositions of *A. roxburghiana*, and some of these variations can be attributed to altitude. The essential oil of *A. roxburghiana* from Bhaldana, Uttarakhand (850 m asl) had β-caryophyllene (18.4%) and eugenol (16.2%) as the major components, while the oil from Bhatwari, Uttarakhand (1218 m asl) had β-caryophyllene (16.3%) and α-thujone (12.0%) as major components [65], and the major components of the essential oil from Mussoorie, Uttarakhand (2205 m asl) were borneol (21.2%), linalyl acetate (7.4%), and α-humulene (6.7%) [65]. Conversely, *A. roxburghiana* oil from Kedarnath, Uttarakhand (3200 m asl) was dominated by β-thujone (65.3%) [45]. *A. roxburghiana*, plants were grown in Garniga, Trento, Italy (800 m asl), from seeds that were collected between 2600 and 4600 in the Kumbu valley of Nepal. The essential oil from these plants were rich in 1,8-cineole (16.6%), camphor (15.2%), and α-thujone (10.0%) [64]. Apparently, there have been no reports on the biological activities of Himalayan *A. roxburghiana* essential oils, and it is difficult to draw any correlations between ethnobotanical use and phytochemical compositions with such wide variations in their compositions.

*Artemisia scoparia* (syn. *A. capillaris*) is widespread and common throughout southwest Asia and central Europe. The aerial parts of *A. scoparia* yield an essential oil with medicinal properties, and has been reported to possess insecticidal, antioxidant, antibacterial, anticholesterolemic, antipyretic, antiseptic, cholagogue, diuretic, purgative and vasodilatatory activities [300]. *A. scoparia* essential oils are generally rich in diacetylenes. Thus, the leaf oil of *A. scoparia* collected from Milam glacier, Uttarakhand, India, was composed of capillene (60.2%), γ-terpinene (11.1%), and 1-phenyl-2,4-pentadiyne (1.0%), while the root essential oil was dominated by capillene (82.9%) and 1-phenyl-2,4-pentadiyne (2.6%) [68]. In contrast, the essential oil from the aerial parts of *A. scoparia* cultivated in New Delhi was composed largely of myrcene (24.4%), γ-terpinene (18.3%), *p*-cymene (17.4%), and neral (12.5%) [324], while *A. scoparia* essential oil from Tajikistan was made up of β-pinene (21.3%), 1-phenyl-2,4-pentadiyne (34.2%), myrcene (5.2%), and capillene (4.9%) [69]. A capillene-rich (42.1%) essential oil of *A. scoparia* from Uttarakhand showed excellent antibacterial activity against *S. aureus* and *B. subtilis* with MIC values of 12.5 μg/mL [59].

Inhabitants of the Nanda Devi National Park, Uttarakhand, India, apply a paste of the leaves of *A. scoparia* on cuts and wounds [67]. The leaf powder is taken to treat diabetes and as a blood purifier, to treat abdominal complaints, colic, cough, and cold. People in the Agra Valley, Parachinar, Pakistan, use the whole plant of *A. scoparia* to treat burns, jaundice, and ear ache; the volatiles of the plant are inhaled for chest congestion [325]. The biological activities of *A. scoparia* and its essential oils are likely due to capillene. This compound has shown antibacterial and antifungal activities [326,327].

*Artemisia vulgaris* is used in Nepal to treat various ailments [70]. The crushed leaves are inserted into the nose to stop bleeding. A bath prepared with the crushed leaves is used to treat allergic reactions. Raw leaves are chewed as a treatment for oral ulcers. In northern Pakistan, the leaf extract of *A. vulgaris* is used to treat malaria and fevers [50]. In Sudhan Gali, Kashmir, Pakistan, an extract of the leaves is used for the treatment of ophthalmic diseases [328]. The leaf essential oil of *A. vulgaris*, collected from Hetauda Makwanpur, Nepal, was found to contain α-thujone (30.5%), 1,8-cineole (12.4%), and camphor (10.3%) [43]. This essential oil was screened for antimicrogial activity against *B. cereus*, *S. aureus*, *E. coli*, *P. aeruginosa*, and *A. niger*, but was found to be inactive (MIC = 2500 μg/mL). Another *A. vulgaris* essential oil sample from Nepal did exhibit antibacterial activity against *Streptococcus pyogenes* and *Propionibacterium acnes* [329].

## 3. The Genus *Cinnamomum*

*Cinnamomum* represents a genus of evergreen aromatic trees belonging to the Lauracaeae comprised of approximately 250 species [299], out of them only eight species have been found in the Nepalese Himalayan region: *C. bejolghota* (Buch.-Ham.) Sweet, *C. camphora* (L.) J. Presl, *C. glanduliferum* (Wall.) Meisn., *C. glaucescens* (Nees) Hand.-Mazz., *C. impressinervium* Meisn., *C. parthenoxylon* (Jack) Meisn., *C. tamala* (Buch.-Ham.) Nees and Eberm., and *C. zeylanicum* Breyn. [330]. This is a very important genus from the aspect of commercial essential oil production.

Traditionally in Nepal, *C. camphora* has been used to treat bronchitis, cold, congestion, diarrhea, dysentery, edema, influenza, flatulence, metabolic and heart problems, as well as various gynecological problems [331]. Five different essential oil chemotypes of *C. camphora* have been identified: (1) camphor, (2) linalool, (3) 1,8-cineole, (4) nerolidol, and (5) borneol [332]. The leaf essential oils of *C. camphora* from Hetauda, central region, Nepal [100], Pantnagar, Uttarakhand, India [97], and Naukuchiatal, Uttarakhand, India [101] were all found to be the camphor chemotype. *C. camphora* leaf oils have shown antifungal activity against *Choanephora cucurbitarum* [99] and antibacterial activity against *Pasturella multocida* [97] and *Aspergillus niger* [100]. In addition to antimicrobial activities, the leaf oil sample from Nepal had shown notable allelopathic activity, cytotoxic activity against MCF-7 human breast tumor cells, and insecticidal activity (*Chaoborus plumicornis*, *Pieris rapae*, *Drosophila melanogaster*, *Solenopsis invicta* × *richteri*) [100]. The traditional use of *C. camphora* to treat bronchitis, colds, and chest congestion is supported by laboratory and clinical investigations. In a Guinea-pig model, camphor vapor was shown to significantly reduce (33%) coughing [333]. A clinical study of topical “vapor rub” containing camphor, menthol, and 1,8-cineole, showed it to be superior to a petrolatum control [334]. In addition, camphor has shown antibacterial activity against the respiratory pathogen *Haemophilus influenza* [335].

People living in the Dolakha district of Nepal apply a paste from the roots of *C. glanduliferum* to treat wounds and toothache [102]. In northern India, leaves of *C. glanduliferum* are used as a stimulant, carminative, and to treat coughs and colds [103]. A leaf oil sample from northern India, rich in 1,8-cineole (41.4%), α-pinene (20.3%), and α-terpineol (9.4%), was found to have antibacterial activity against Gram-positive bacteria (*Micrococcus luteus*) and Gram-negative bacteria (*Escherichia coli*, *Pseudomonas aeruginosa*, and *Aeromonas salmonicida*). The high concentration of 1,8-cineole likely contributes to its efficacy against coughs and colds. 1,8-Cineole has shown clinical efficacy as a mucolytic and spasmolytic as well as beneficial effects in inflammatory airway diseases such as asthma and chronic obstructive pulmonary disease (COPD) [336,337]. The antibacterial activity of *C. glanduliferum* leaf oil is likely not due to 1,8-cineole alone [338], but may be attributed to synergistic effects between 1,8-cineole and other minor components [339,340]. Another chemotype of *C. glanduliferum*, rich in (*E*)-nerolidol (52.2%), has been reported, but no biological activities were investigated for this oil [107]. (*E*)-Nerolidol has shown antibacterial activity, however [341,342].

*C. glaucescens*, commonly known as “sugandhwal kokila”, has been traditionally used as demulcent and stimulant and has shown analgesic, antiseptic, astringent, and carminative properties [343]. Seeds of *C. glaucescens* are used for treatment of common cold, cough, toothache and taenias; the seed paste is applied to treat muscular swellings; the seed oil has also been demonstrated to treat muscular spasm, joint pain and body aches. [344]. In Manipur, India, the powdered bark is used to treat kidney trouble [104]. The fruit essential oil of *C. glaucescens* from Nepal was dominated by methyl (*E*)-cinnamate (40.5%) [100], whereas a commercial fruit essential from Nepal had methyl (*E*)-cinnamate (14%) 1,8-cineole (13%), and α-terpineol (7%) as the major components, while the pericarp oil was rich in 1,8-cineole (56%) [106]. The essential oil obtained from fruits from Lucknow, India, was also rich in 1,8-cineole (43.6%) [105]. In comparison, the leaf oil of *C. glaucescens* from northeast India contained elemicin (92.9%) and methyl eugenol (4.9%) as major components [107]. The fruit essential oil from Nepal showed nematicidal (*Caenorhabditis elegans*) and insecticidal (*Culex pipiens*, *Reticulitermes virginicus*) activity [100], while the fruit oil from Lucknow was insecticidal (*Callosobruchus chinensis*) and antifungal (*Aspergillus flavus*) [105]. The nematicidal activity of *C. glaucescens* fruit oil is consistent with the traditional use of the plant to expel tapeworms. Methyl (*E*)-cinnamate was shown to be active against *C. elegans*, but 1,8-cineole was not [100].

*Cinnamomum tamala* leaf essential oil has shown some variation in composition. Cinnamaldehyde is generally a major component [97,101,108], but a leaf oil sample from Pannagar, Uttarakhand was dominated by eugenol (65%) [97]. By contrast, *C. tamala* leaf oil from Karachi, Pakistan, was composed largely of β-caryophyllene (25.3%), linalool (13.4%), and caryophyllene oxide (10.3%) [345]. In far-western Nepal, leaves of *C. tamala* are used to treat gastic problems [10], while in the Kathmandu area of Nepal, the leaves are used as a spice and flavoring agent [42]. The leaf oil from Uttarakhand has shown activity against foodborne bacteria, *Salmonella enterica*, *Escherichia coli*, and *Pasturella multocida* [97]. A leaf oil sample from Jharkhand, India, demonstrated antifungal activity against *Aspergillus niger*, *Aspergillus fumigatus*, *Candida albicans*, *Rhizopus stolonifer*, and *Penicillium* spp., but the composition of the oil was not reported [346].

## 4. The Genus *Cymbopogon*

Aromatic grasses are one of the chief sources of essential oils. The genus *Cymbopogon* is comprised of about 140 species worldwide, out of which 45 species have been reported to occur in India. *Cymbopogon* is one of the most important essential oil yielding genera of the family Poaceae [347,348,349]. The most common economic species *viz.*, *C. winterianus* Jowitt ex Bor, *C. flexuosus* (Nees ex Steud.) Will. Watson, *C. martinii* var. *motia* Bruno, *C. martinii* var. *sofia* Bruno, *C. nardus* var. *nardus* (L.) Rendle, *C. citratus* (DC.) Stapf, *C. pendulus* (Nees ex Steud.) Will. Watson, *C. jwarancusa* (Jones) Schultz, and *C. khasianus* (Munro ex Hack.) Stapf ex Bor, produce different types of essential oils, such as palmarosa oil (*C. martinii* var. *motia*), lemongrass oil (*C. citratus*, *C. flexuosus*), citronella oil (*C. winterianus*, *C. nardus*), ginger grass oil (*C. martinii* var. *sofia*), or rusa oil (*C. martinii* var. *motia*) of commercial interest [350,351,352]. Three *Cymbopogon* grasses, namely, Java citronella (*C. winterianus*), East Indian lemongrass (*C. flexuosus* and *C. pendulus*) and palmarosa (*C. martinii* var. *motia*) are the most common species that are widely cultivated for their essential oils of commercial importance used in perfumes, soaps, cosmetics, toiletry, tobacco products and other related industrial products [353,354]. In India, the total area under cultivation of these aromatic grasses is more than 40 thousand hectares, distributed mainly in Assam, Kerala, Madhya Pradesh, South Gujarat, Karnataka, Maharashtra, Andhra Pradesh and Uttar Pradesh [355,356,357,358]. Several *Cymbopogon* species have demonstrated considerable anthelmintic, anti-inflammatory, analgesic, anti-ageing, pesticidal, antimicrobial, mosquito repellant, and larvicidal activities and thus, are used in native medicine for curing a number of diseases [350,359,360]. The *Cymbopogon* species have great prospects for producing quality essential oils [359,360], and it has direct relevance to the perfumery industry with economic benefit to humankind [361,362].

Lemongrass oil is distilled from two morphologically different species of lemongrass, *C. flexuosus* (common name: East Indian lemongrass) and *C. citratus* (common name: West Indian lemongrass). Geraniol (30.5%), citronellol (24.1%), neral (10.3%), and geranial (13.6%) have been reported as the major components of *C. flexuosus* [363], but many chemotypes / cultivars / variants have been reported for *C. flexuosus* [364,365,366,367,368,369,370,371,372,373]. The oil of lemongrass is widely used in soaps and detergents [374]. The antifungal, antibacterial, and antioxidant properties of lemongrass oil have been widely utilized [59,374,375,376,377,378,379,380,381].

The North Indian lemongrass oil (*C. pendulus*) occurs in wild areas of northern India such as Saharanpur (in the state of Uttar Pradesh) [382] and western Nepal [383], and is generally rich in geranial (48%) and neral (33%), with lesser amounts of geraniol (5%) and linalool (3%) [358]. Palmarosa oil, distilled from *C. martinii* var. *motia*, has geraniol as the major component (71%–89%) [384] and is considered better in quality [385,386]. The essential oil produced from the *sofia* variety of *C. martinii* Stapf is known as gingergrass oil. The *cis* and *trans* forms of *p*-menth-2,8 diene-1-ol, *p*-menth1(7),8 dien-2-ol, carveol, and piperitol, along with limonene (20%) and monoterpene alcohols, have been reported from the wild strain of *C. martinii* var. *sofia* growing in Kumaon hills [355,385]. A new hemiacetal bis monoterpenoid compound cymbodi acetal was characterized in the oil of *C. martinii* [387].

The leaf essential oil from *C. jwarancusa* (Jones) Schult. is rich in piperitone, imparting a characteristic odor [388]. The major components in *C. jwarancusa* oil are piperitone (45%–67%) and elemol (7%–29%) [389,390,391,392].

The components of the essential oils of *C. distans* differ with growth conditions and geographical locations [393]. Thus, for example, the essential oil from Munsyari (Uttarakhand) was composed of citral (neral + geranial) (35.0%), geranyl acetate (15.0%), and geraniol (9.5%) [122]. Similarly, the essential oil cultivated in Pantnagar, Uttarakhand was made up predominantly of geranial (22.8%), neral (16.9%), geraniol (14.8%), and geranyl acetate (19.5%) [394]. However, the oil from Nainital (Uttarakhand) was dominated by α-oxobisabolene (68%) [122], while *C. distans* var. Loharkhet essential oil was rich in the sesquiterpenoids eudesmanediol (34.4%) and 5-*epi*-7-*epi*-α-eudesmol (11.2%) [395]. Mathela and co-workers had recognized four chemotypes of *C. distans* from the Kumaon and Garhwal regions of Uttar Pradesh (India) having marker compounds α-oxobisabolene (chemotype I); citral, geraniol, and geranyl acetate (chemotype II); piperitone, limonene, and eudesmanediol (chemotype III); and sesquiterpene alcohols (chemotype IV) in their oils [396]. A study carried out by Lohani and co-workers [123] revealed three additional distinct chemotypes: Chemotype I (*p*-menth-2-en-1-ol, piperitol, α-terpinene), chemotype II (borneol, bornyl acetate, limonene), and chemotype III (piperitone, α-terpinene), to give a total of seven different chemotypes for *C. distans*.

## 5. The Genus *Juniperus*

There are around 75 species of *Juniperus* (Cupressaceae), and is a very diverse genus ranging in habitat from sea level to above timberline [397]. Important medicinal species include *J. communis*, the common juniper used to flavor gin [398], *J. drupacea* from the eastern Mediterranean [399], *J. monosperma* from southwestern North America [400], *J. oxycedrus*, the heartwood from which oil of cade is prepared [401], and *J. virginiana*, used in traditional medicine by Native Americans in eastern North America [402].

In the Himalaya of Nepal and northern India, there are at least six species of native *Juniperus*: *J. communis* L., *J. indica* Bertol., *J. macropoda* Boiss (syn. *J. excelsa* M. Bieb.), *J. pseudosabina* Fisch. and C.A. Mey., *J. recurva* Buch.-Ham. Ex D. Don (syn. *J. squamata* Lamb.), and *J. wallichiana* Hook. f. and Thomson ex E. Brandis [397,403,404,405,406]. *J. communis* is the most widespread species of *Juniperus* and is distributed circumpolar, including the Himalayas from Kashmir to Bhutan [407]. *J. communis* is used in traditional medicine throughout the Himalayas. For example, the local people in Kishtwar, Jammu and Kashmir, India, apply the oil extracted from the plant to treat rheumatism [182]. Similarly, inhabitants of Parvati valley in Himachal Pradesh use an extract from the twigs to treat joint pain [56]. Essential oils of *J. communis* are rich in α-pinene and limonene [149], and both α-pinene [408,409] and limonene [410] have shown antinociceptive effects in rodents, consistent with the ethnobotanical use of *J. communis* to treat joint pain. In addition to using the plant for gout, chronic arthritis, and rheumatism, *J. communis* is taken as a tonic, diuretic, for urinary tract infection [411], and a paste made from the leaves is applied to skin ailments [67]. Essential oils from the berries of *J. communis* have shown antifungal (*Candida albicans*, *Candida kefyr*, *Epidermophyton floccosum*, *Trichophyton rubrum*, *Trichophyton mentagrophytes*, *Trichophyton rubrum*, *Microsporum canis*) and antibacterial (*Bacillus cereus*) activity [146,147,148], which is consistent with its use to treat urinary tract infection and skin infections.

In the Humla district of western Nepal, a decoction of the leaves and berries of *Juniperus indica* are consumed to treat coughs and colds; a paste of the berries is applied externally to cure skin diseases [44]. Similarly, inhabitants in Upper Mustang, Nepal, use the fruits and leaves of *J. indica* for skin diseases, fevers, and coughs [150]. The leaf and berry essential oils of *Juniperus indica* are generally rich in sabinene and terpinene-4-ol [149,151,152]. Terpinen-4-ol has shown antibacterial activity against several bacteria, including methicillin-resistant *Staphylococcus aureus* (MRSA) [412], respiratory tract pathogens *Haemophilus influenza*, and penicillin-resistant *Streptococcus pneumoniae* [413]. In addition, terpinen-4-ol been shown to inhibit the growth of human melanoma (M14 WT) cells [414]. Terpinen-4-ol has shown antifungal activity against several dermatologically important fungi, including *Candida albicans* (responsible for cutaneous moniliasis), *Candida parapsilosis* (responsible for onychomycosis), and several dermatophytes responsible for tinea in humans (*Trichosporon* spp., *Rhodotorula rubra*, *Epidermophyton floccosum*, *Microsporum canis*, and *Trichophyton mentagrophytes*); the compound was also active against the potential pulmonary fungal pathogens *Aspergillus niger*, *Aspergillus flavus*, and *Aspergillus fumigatus* [415]. Thus, the biological activities of terpinene-4-ol in *J. indica* oils are consistent with the ethnobotanical uses of the plant for respiratory and dermal infections.

In Himachal Pradesh, the berries of *Juniperus macropoda* are used to treat colic, cough, chest colds, diarrhea, impotency, and indigestion; the resin is used externally on ulcers [152]. In the Ladakh range in northern Jammu and Kashmir [153,416] and in Tibet [417], the needles are used as incense. In Tibet, the needles are used medically to treat kidney diseases [417], while in Ladakh, tablets prepared from the wood is used for irregular menstrual cycles, amenorrhea or dysmenorrhea [418] and tablets made from *J. macropoda* berries, mixed with several other plants, are taken for kidney and urinary disorders [419]. The leaf essential oils of *J. macropoda* have shown wide variation in chemical composition. A sample of leaf oil from Chamba, Himachal Pradesh had sabinene (27.5%), cedrol (14.1%), and terpinen-4-ol (9.4%) as the major components [154]. This oil did show antifungal activity and mosquito larvicidal activity. A leaf oil sample from Hindokhal, Uttarakhand, was dominated by β-elemene (42.5%) *trans*-sabinene hydrate (8.8%), and α-cubebene (7.9%) [156], while another sample, from Mussorie, Uttarakhand, was rich in α-thujone (22.6%), biformene (7.7%), sabinene (5.8%) [156]. Unfortunately, there do not seem to have been any phytochemical investigations on *J. macropoda* from Kashmir.

There do not seem to be any published reports on ethnopharmacological uses of *Juniperus pseudosabina*. *J. recurva*, on the other hand, is used in Nepal. Thus, the local people in the Rasuwa district of central Nepal use *J. recurva* to treat fever, headache, coughs, and colds [11]; the local people in the Humla district of northwestern Nepal, apply a paste of the leaves and berries to treat skin conditions [44]. In the Nubra River valley of northern Jammu and Kashmir, the people use a decoction of the leaves of *J. recurva* to lower fever in children [7]. Leaf essential oils of *J. recurva* are rich in δ-3-carene [157], but there have apparently been no bioactivity studies on *J. recurva* essential oils.

## 6. The Genus *Nepeta*

*Nepeta* (Lamiaceae) is a genus of about 250 species of flowering herbs, small shrubs, rarely trees, often with quadrangular stems, glandular and aromatic, with opposite leaves placed successively at right angles to each other [420]. Among 31 species reported in the Himalayan region, six are found in the Kumaun region of Uttarakhand: *N. ciliaris* Benth., *N. connata* Royle ex Benth., *N. distans* Royle ex Benth., *N. elliptica* Royle ex Benth., *N. leucophylla* Benth., and *N. spicata* Wall ex Benth. [421]. Eleven species of *Nepeta* are native to Nepal: *N. cataria* L., *N. ciliaris*, *N. coerulescens* Maxim., *N. discolor* Royle ex Benth., *N. elliptica*, *N. hindostana* (Roth) Haines, *N. laevigata* (D. Don) Hand.-Mazz., *N. lamiopsis* Benth. ex Hook. f., *N. leucophylla*, *N. nepalensis* Spreng., and *N. staintonii* Hedge [330]. In addition, *N. campestris* Benth. and *N. eriostachys* Benth. are endemic to Kashmir, India [422].

*Nepeta* species are used traditionally as antispasmodic, diuretic, febrifuge, diaphoretic, antimicrobial and antiseptic agents and also in the treatment of dysentery, tooth trouble and kidney and liver diseases [423]. Diverse biological activities, e.g., feline attractant [424], insect repellant [425], and arthropod defense [426,427] are attributed to the presence of biologically active iridoids, monoterpene nepetalactones, in *Nepeta* species [428]. Aydin *et al.* investigated the antinociceptive effects of essential oils from *Nepeta* species, including *N. phyllochlamys*, *N. nuda* ssp. *nuda*, and *N. caesarea*, using a tail flick and tail immersion tests [429]. These authors detected central and peripheral antinociceptive effects in *N. caesarea* and concluded that 4aα,7α,7aα-nepetalactone was the active principle and had a specific opioid receptor subtype agonistic activity.

*Nepeta* species are used in the traditional medicine of many cultural groups in the Himalayas. Many species are used to reduce fever, treat coughs and colds, and relieve digestive disorders (Table 1). Nepetalactones are generally considered biochemical markers for the genus and some some Himalayan *Nepeta* essential oils are rich in nepetalactones, e.g., *N. elliptica* [200] and *N. juncea* [210]. The antimicrobial activities of these essential oils are likely due to nepetalactone concentrations [430,431,432]. Nevertheless, many Himalayan *Nepeta* samples contain little or no nepetalactones [216], and therefore, the ethnomedicinal uses and biological activities observed in these *Nepeta* species are likely due to other constituents.

Some *Nepeta* spp. have large concentrations of 1,8-cineole, *viz. N. discolor* [200], *N. laevigata* [214], and *N. royleana* [217]. Although 1,8-cineole has been shown not to have antitussive activity [333,433], the compound has shown efficacy in acute rhinosinusitis and alleviate headache, nasal obstruction, and rhinological secretion in a double-blind, placebo-controlled study [434]. In addition, 1,8-cineole has demonstrated ulcer-healing and gastroprotective properties in rats [435] as well as antispasmodic effects on isolated mouse ileum [436]. Several other *Nepeta* samples have been rich in sesquiterpenoids such as β-caryophyllene [200,204,209,214,218], caryophyllene oxide [201,214,218], and germacrene D [200,204,209,218] (see Table 1). β-Caryophyllene has shown anesthetic [437], anti-inflammatory activity [438], but not analgesic activity [439], in animal models. The compound ameliorated colitis in a mouse model [440,441] and has shown antispasmodic effects on isolated rat ileum [442]. Caryophyllene oxide has shown analgesic as well as anti-inflammatory activity in mice [443].

## 7. The Genus *Origanum*

The members of the genus *Origanum* L. are usually perennial herbs belonging to the mint family (Lamiaceae). It has been classified into 10 sections including 43 species, 6 subspecies, 3 varieties and 18 naturally occurring hybrids, widely distributed in the Mediterranean, Euro-Siberian and Irano-Siberian regions [444,445]. Members of the genus are mainly distributed along the Mediterranean region, with 75% restricted to the eastern Mediterranean [446]. The genus includes some commercially important culinary herbs, including oregano (*Origanum vulgare* L.) and marjoram (*Origanum majorana* L., syn. *Majorana hortensis* Moench), which are extensively used for flavoring food products and alcoholic beverages. In India and Nepal, only one species is available from sub-tropical to alpine zones of the Himalayan Region [6].

*Origanum vulgare* commonly is known as “oregano” in most European countries and “Himalayan marjoram” or “Indian oregano” in India. This is the only species reported from northwestern Himalaya, found in an altitude between 600 and 4000 m of Kumaon and Garhwal region of Uttarakhand Himalaya [226]. There are numerous chemotypes of *O. vulgare*, and Verma and co-workers have defined six in Himalayan India [229]: (1) γ-terpinene/thymol, (2) thymol/ocimene, (3) thymol/γ-terpinene, (4) γ-terpinene/carvacrol, (5) carvacrol/γ-terpinene, and (6) linalool. Lukas and co-workers have generalized European *O. vulgare* monoterpene chemotypes as (a) cymyl-type (rich in *p*-cymene, thymol, and/or carvacrol), (b) acyclic-type (rich in myrcene, ocimene, linalool and linalyl derivatives), and (c) sabinyl-type [447].

The thymol- and carvacrol-rich chemotypes of *O. vulgare* should be useful in treating bronchial and pulmonary diseases (coughs, colds, *etc.*); both thymol and carvacrol are antibacterial [448], antitussive [449,450], antihistamine [451], and numerous other pharmacological properties [452], which are consistent with traditional uses of this plant. The monoterpenoid alcohols, linalool, terpinen-4-ol, and α-terpineol [338,453], the sesquiterpenoids β-caryophyllene, α-humulene, and germacrene D [454] have also shown antimicrobial effects, consistent with the potential activities and uses of the other chemotypes of *O. vulgare*.

## 8. The Genus *Valeriana*

*Valeriana* L. (Caprifoliaceae) consists of around 200 species distributed in the temperate and sub-tropical areas globally and is among the important herbal traditional drug in various pharmacopeias [299]. The herbal drug valerian consists of the subterranean organs (rhizome, root, stolons) of *Valeriana officinalis* L. [172]. The valerian-derived phytomedicines have been used for curing nervous unrest, emotional troubles (as tranquillizer/sedative), epilepsy, insanity, snake envenomation, eye-trouble, skin-diseases, relaxant, carminative, and for improving the complexion [455,456,457]. Valerian is one of the top ten selling herbal supplements in North America [458]. It has also been prescribed as the perfect herbal tranquilizer, and was used for this purpose in the First World War to treat soldiers suffering from shell shock and to calm civilians subjected to air raids during World War II [459].

In India, *Valeriana jatamansi* Jones (syn. *Valeriana wallichii* DC.). has long been used in Ayurveda and Unani systems of medicine, which describe its use in skin diseases, insanity, epilepsy, and snake bite, and is considered to have remarkable sedative effects in nervous unrest, stress, and neuralgia [460,461]. A survey of the literature has revealed the presence of flavonoid glycosides [462,463], iridoids, and lignans [464,465,466] in *V. jatamansi*. Anti-inflammatory [467] antianxiety [468], antidiarrheal, and bronchodilatory activities [469] of *V. jatamansi* extracts have been scientifically validated. The plant has also shown *in vitro* cytotoxic [470] and antileishmanial [471] activities. *V. jatamansi* essential oil has shown antimicrobial activity against pathogenic bacteria and as well as antifungal activity against different human and plant fungal pathogens [472].

The chemical compositions of root/rhizome essential oils show six chemically distinct chemotypes within *V. jatamansi.* (a) a maaliol-rich (~ 40%–60%) chemotype [287,288], (b) a patchouli alcohol-rich (> 40%) chemotype [288,289,290,291], (c) a patchouli alcohol/α-bulnesene chemotype [289,291], (d) a patchouli alcohol/viridiflorol chemotype [290], (e) a seychellene-rich chemotype [288], and (f) a kanokonyl acetate chemotype [292]. The root oil of *V. himalayana* from Talle valley of Arunachal Pradesh was mainly composed of methyl linoleate, valeracetate, bornyl acetate, and cuparene [279]. The roots of *V. hardwickii* var. *arnotiana* revealed constituents belonging to two different chemotypes [282]: Chemotype I, collected from an altitude of 3500 m from Milam glacier contained valeracetate, 8-epikessyl glycol diacetete, α-kessyl acetate, and malliol as the marker compounds, while chemotype II, collected from Vishnu Prayag, contained kessanyl acetate and maaliol as the main constituents. Epoxysesquithujene, a novel sesquiterpenoid, was isolated from *V. hardwickii* var. *hardwickii* [280]. The main constituents of root oil of *V. pyrolaefolia* were valeranone and patchouli alcohol [473].

## 9. Conclusions 

The Himalayas, with wide-ranging elevations, deep glacial and river valleys, areas of high rainfall and areas of high desert, is a rich area of biodiversity with much endemism. Traditional herbal medicine continues to play a role in many tribal areas, and numerous medicinal plants and their essential oils have shown remarkable biological activities. Unfortunately, there remains a paucity of information relating biological activities of essential oils with the ethnobotanical uses of the plants. In many cases this may be due to the activity residing in non-volatile components. Additionally, many phytochemical researchers have neglected bioactivity screening related to ethnopharmacological uses. Thus, there is much additional work that can be carried out to identify phytochemicals associated with biological activities that support traditional uses of medicinal plants. In addition, several aromatic plants have shown commercial promise as flavoring agents, fragrances, cosmetics, and pesticides. Due, in part, to the great demand for essential oils, herbal medicines, and pharmaceuticals, the medicinal plants of the Himalayas are threatened by unsustainable harvesting [474], and increasing environmental degradation, invasive plant species, and climate change also threaten Himalayan native flora. We encourage the preservation of traditional knowledge and uses of Himalayan medicinal plants and we hope that additional steps are undertaken to protect and maintain the Himalayan ecology.

## Figures and Tables

**Table 1:** Ethnopharmacology, biological activities, and essential oil compositions of Himalayan aromatic medicinal plants.

Plant species (Family)	Ethnopharmacology	Bioactivity of Himalayan Essential Oil	Major essential oil components
*Abies pindrow* (Royle ex D. Don) Royle (Pinaceae)	The tribal people of the Sewa River area of Jammu and Kashmir, India, use the leaves to treat bronchitis and asthma; the inner bark is taken for constipation; and the cones are used as a diuretic and purgative [7].	None reported for Himalayan essential oils.	Leaf essential oil from Uttarakhand, India: α-pinene (16.8%), camphene (19.9%), β-pinene (6.5%), myrcene (6.7%), limonene (21.0%) [8]. Stem essential oil from Uttarakhand: α-pinene (13.8%), β-pinene (8.6%), myrcene (8.3%), limonene (24.4%) [8].
*Achillea millefolium* L. (Asteraceae)	The tribal people of the Sewa River area of Jammu and Kashmir, India, use an infusion of plant as a diuretic; vapors from leaves and flowers are used to treat colds and fever; tea from leaves is given to treat cold [7].	None reported for Himalayan essential oils.	Aerial parts essential oil from Srinagar, Kashmir (Jammu and Kashmir, India): β-pinene (10.6%), 1,8-cineole (15.1%), β-caryophyllene (16.2%), α-terpineol (0.1%), borneol (0.2%) [9]. Aerial parts essential oil from Sisso, Lahaul-Spiti (Himachal Pradesh, India): β-pinene (14.0%), 1,8-cineole (3.2%), β-caryophyllene (12.5%), α-terpineol (4.4%), borneol (8.5%) [9].
*Acorus calamus* L. (Araceae)	The tribal people of the Sewa River area of Jammu and Kashmir, India, apply the leaf paste to wounds [7]. The people of Baitadi and Darchula districts of far-western Nepal use the juice of the rhizome as an anthelmintic; the juice is given for stomachache [10]. In the Rasuwa District of central Nepal [11], and the Seti River area of western Nepal [12], the rhizome is chewed to treat coughs, colds, and sore throat. In the Jutpani Village, Chitwan district of central Nepal, the rhizome paste is applied to wounds and swelling to reduce inflammation [13].	Rhizome oil from Biratnagar, Nepal. *Artemia salina* lethality (LC_50_ = 9.5 μg/mL), cytotoxic (MCF-7), antifungal (*Aspergillus niger*, MIC = 19.5 μg/mL) [14]. (*Z*)-Asarone from *Acorus calamus* inhibited growth of *Candida albicans* at 0.5 mg/mL and was fungicidal at 8 mg/mL [15]. (*Z*)-Asarone inhibited intracellular lipid accumulation during adipocyte differentiation [16].	Rhizome essential oil from Biratnagar, eastern Nepal. Rhizome: (*Z*)-asarone (84.0%–86.9%), (*E*)-asarone (1.9%–4.0%) [14]. Leaf essential oil from Biratnagar, Nepal: (*Z*)-asarone (78.1%), (*E*)-asarone (9.9%) [14]. Rhizome oil from Uttarakhand, India: (*Z*)-asarone (81.1%–92.4%) [17].
*Aegle marmelos* (L.) Corrêa (Rutaceae)	The people of Baitadi and Darchula districts of far-western Nepal use a leaf decoction used to treat dysentery, diarrhea, respiratory tract infections, and heart ailments [10]. Tribal people in the Seti River area of western Nepal use the bark juice against diarrhea and stomachache [12]. In India, the leaf paste is used externally to treat abcesses, cuts, wounds, ulcers. Fruit is taken internally for gastrointestinal problems (vomiting, dysentery, diarrhea) [18]. People in Kumaun, Uttarakhand, use the fruits to treat digestive disorders [19].	Leaf oil from from Biratnagar, eastern Nepal, *Culex pipiens* larvicidal (LC_50_ = 2.15 μg/mL), *Caenorhabditis elegans* nematicidal (LC_50_ = 113 μg/mL), insecticidal (*Reticulitermes virginicus*, *Drosophila melanogaster*, *Solenopsis invicta* × *richteri*) [20].	Leaf essential oil from Biratnagar, eastern Nepal: limonene (64.1%), (*E*)-β-ocimene (9.7%), germacrene B (4.7%) [20]. Several leaf oil samples from Uttarakhand, India: limonene (31.0%–90.3%), α-phellandrene (trace-43.5%), (*E*)-β-ocimene (0.7%–7.9%) [21].
*Ageratum conyzoides* L. (Asteraceae) *	Tribal people in the Seti River area of western Nepal apply the leaf juice to cuts and wounds [12]. People from Kumoun, Uttrakhand, India use the leaf extract to stop bleeding [19] and to treat skin diseases (ringworm, scabies, sores, burns boils, cuts) [22].	None reported for Himalayan essential oils.	Aerial parts essential oil from Kumaun, Uttarakhand, India: ageratochromene (42.5%), demothoxyageratochromene (16.7%), β-caryophyllene (20.7%) [23].
*Ageratum houstonianum* Mill. (Asteraceae) *	Plant juice used externally to treat cuts and wounds [24].	Aerial parts essential oil from India, antibacterial (*Micrococcus luteus*, *Rhodococcus rhodochrous*) [25].	Aerial parts essential oil from India: ageratochromene (52.6%), demothoxyageratochromene (22.5%), β-caryophyllene (9.7%) [25].
*Ajuga parviflora* Benth. (Lamiaceae)	Tribal people in the Mornaula Reserve Forest of Kumoun, west Himalaya, India use the leaves used as an anthelmintic (*Ascaris*) [22].	None reported for Himalayan essential oils.	Leaf essential oil from Uttarakhand, India: β-caryophyllene (22.4%), γ-muurolene (12.7%), γ-terpinene (6.3%), caryophyllene oxide (6.2%) [26].
*Amomum subulatum* Roxb. (Zingiberaceae)	In Ayurveda, the plant is used to treat indigestion, vomiting, biliousness, abdominal pains, rectal diseases; throat trouble, lung congestion, pulmonary tuberculosis [27].	Seed oil from Terhathum district, eastern Nepal, antifungal (*Aspergillus niger*, MIC = 19.5 μg/mL), nematicidal (*Caenorhabditis elegans*, LC_50_ = 341 μg/mL), insecticidal (*Drosophila melanogaster*, LC_50_ = 441 μg/mL) [28].	Seed essential oil from Terhathum district, eastern Nepal: 1,8-cineole (60.8%), α-terpineol (9.8%), α-pinene (6.4%), β-pinene (8.3%). Pericarp: 1,8-cineole (39.0%), α-terpineol (12.3%), α-pinene (4.8%), β-pinene (17.7%) [28]. Several seed oil samples from Himachal Pradesh, India: 1,8-cineole (50.6%–60.5%), α-terpineol (14.9%–16.5%), limonene (5.5%–11.8%), terpinen-4-ol (2.6%–5.4%), nerolidol (3.8%–6.0%) [29].
*Anisomeles indica* (L.) Kuntze (Lamiaceae)	In the Mornaula Preserve Forest of Kumoun, west Himalaya, the people use the whole plant as an antidote to poisonous bites [22]. In far western Nepal, the leaf extract is taken for urinary complaints [30].	Leaf oil from Toranmal Forest, Satpuda Valley, Maharashtra, India, antibacterial (*Bacillus pumilus*) [31].	Leaf essential oil from Toranmal Forest, Satpuda Valley, Maharashtra, India: isobornyl acetate (64.6%), isothujone (6.0%) [31].
*Aralia cachemirica* Decne. (Araliaceae)	The root is used traditionally in Himachal Pradesh, India, for gastric complaints [32].	None reported for Himalayan essential oils.	Leaf essential oil from Uttarakhand, India: α-pinene (41.0%), β-pinene (35.1%). Root: α-pinene (52.7%), β-pinene (13.6%) [33].
*Aristolochia indica* L. (Aristolochiaceae)	Used in traditional medicine in India [34]. The root, leaf, stem bark, given for fever, as an anthelmintic (intestinal worms), and to treat snakebites. Given to children to treat diarrhea and bowel complaints.	None reported for Himalayan essential oils.	Stem essential oil from Arunachal Pradesh: *trans*-pinocarveol (24.4%), α-pinene (16.4%), pinocarvone (14.2%) [35].
*Artemisia dracunculus* L. (Asteraceae)	*A. dracunculus* (tarragon) is used throughout the world flavoring food [36,37]. In the Nubra Valley (Kashmir), Kibber Wildlife Sanctuary (Himachal Pradesh), and the Lahual Valley (Himachal Pradesh), an extract of the plant is used used to relieve toothache, reduce fever, and as a treatment for gastrointestinal problems [38,39,40].	None reported for Himalayan essential oils.	Aerial parts essential oil from Shansha, Kirting (Himachal Pradesh), India: capillene (58.4%), (*Z*)-β-ocimene (8.6%), β-phellandrene (7.0%), terpinolene (5.9%) [36]. Leaf oil sample from Sanat Nagar, (Jammu and Kashmir), India: acenaphthene (51.7%), capillene (12.6%), (*Z*)-β-ocimene (12.2%). Stem: acenaphthene (32.6%), capillene (34.7%), (*Z*)-β-ocimene (17.6%). Root: acenaphthene (66.6%), capillene (22.8%) [37]. Aerial parts essential oil from Kashmir, India: capillene (60.2%), (*Z*)-β-ocimene (12.7%), 5-phenyl-1,3-pentadiyne (5.1%) [41]
*Artemisia dubia* Wall. ex Besser (Asteraceae)	In Newar community of Kathmandu, Nepal, the leaf juice is used to treat cuts and wounds [42].	Leaf oil sample from Kirtipur, Kathmandu, Nepal showed cytotoxic (MCF-7) activity and marginal antifungal activity (*Aspergillus niger*) [43].	Leaf oil sample from Kirtipur, Kathmandu, Nepal: chrysanthenone (29.0%), coumarin (18.3%), and camphor (16.4%) [43].
*Artemisia gmelinii* Weber ex Stechm. (Asteraceae)	In the Humla district of northwestern Nepal, the fresh plant is ground into a paste an applied externally to cure headache, boils, and pimples [44].	None reported for Himalayan essential oils.	Aerial parts essential oil from a sample from Malari, Garhwal region, India: artemisia ketone (28.2%), 1,8-cineole (13.0%), sabinene (6.6%) [45]. Essential oil from the aerial parts of a sample from Niti valley, Uttarakhand, India: artemisia ketone (53.3%), α-thujone (9.9%), 1,8-cineole (6.6%) [46]. Essential oil from the aerial parts of a sample from Jhelum, Uttarakhand, India: artemisia ketone (40.9%), α-thujone (4.0%), *ar*-curcumene (8.5%) [46].
*Artemisia indica* Willd. (Asteraceae)	In the Rasuwa District of central Nepal [11], and the Seti River area of western Nepal [12], a leaf paste is applied to cuts and wounds. In the Newar community of Kathmandu, Nepal, the whole plant/leaf juice is used for anti-leech and indigestion [42].	Sample from Nepal not antimicrobial; not cytotoxic [43]. Sample from Kashmir antibacterial (*Bacillus subtilis*, *Staphylococcus epidermidis*, *Pseudomonas aeruginosa, Salmonella typhi*, *Klebsiella pneumoniae*, *Penicillium chrysogenum*, *Aspergillus niger*) and cytotoxic [THP-1 (leukemia), A-549 (lung), HEP-2 (liver) and Caco-2 (colon)] [47]	Leaf oil sample from Dhulikhel, Kavre, Nepal: ascaridole (15.4%), isoascaridole (9.9%), *trans-p*-mentha-2,8-dien-1-ol (9.7%), and *trans*-verbenol (8.4%) [43]. Aerial parts essential oil from Daksum, Kokerrnag (Kashmir), India: artemisia ketone (42.1%), germacrene B (8.6%), borneol (6.1%) and *cis*-chrysanthenyl acetate (4.8%) [47]. Aerial parts essential oil from Garhwal Himalaya, Uttarakhand, India: davanone (30.8%), β-pinene (15.3%), germacrene d (5.8%) [48].
*Artemisia japonica* Thunb. (Asteraceae)	In the Garhwal Himalaya (Uttarakhand), the leaves used as incense and insecticide [49]. In northern Pakistan, the leaf extract used to treat malaria; paste of leaves used externally on skin diseases [50].	None reported for Himalayan essential oils.	Aerial parts essential oil from Uttarakhand: linalool (27.5%), germacrene d (11.2%), (*E*)-β-ocimene (6.5%), 1,8-cineole (5.5%), (*Z*)-β-ocimene (5.5%) [51].
*Artemisia maritima* L. (Asteraceae)	The Bhots people of Spiti Valley, Himachal Pradesh, India apply the root juice externally to treat boils; a decoction of the leaves is taken orally to remove abdominal parasites [52].	The essential oil from Lahaul-Spiti, Himachal Pradesh, not antimicrobial [53].	Aerial parts essential oil from a sample from Malari, Garhwal region, India: α-thujone (63.3%), sabinene (7.8%), 1,8-cineole (6.5%) [45]. Aerial parts essential oil from Pooh, Himachal Pradesh, India: 1,8-cineole (23.8%), chrysanthenone (17.5%) [54]. Aerial parts essential oil from Rhongtong Pass, Himachal Pradesh, India: 1,8-cineole (37.3%), chrysanthenone (38.1%) [54]. Aerial parts essential oil from Lahaul-Spiti, Himachal Pradesh, India: 1,8-cineole (44.2%), camphor (9.2%), borneol (10.9%) [54]. Essential oil from the aerial parts growing in Chamoli district of Garhwal (Uttarakhand), India: 1,8-cineole (23.6%), chrystanthenone (25.7%), germacrene D (6.7%) [55]. Aerial parts essential oil from Lahaul-Spiti, Himachal Pradesh, India: 1,8-cineole (27.2%), camphor (44.4%), camphene (5.9%) [53].
*Artemisia nilagirica* (C.B. Clarke) Pamp. (Asteraceae)	People in the Parvati valley (Himachal Pradesh), India, apply a leaf paste to cuts and wounds [56]. In Darjeeling (West Bengal) India, the plant is chewed to treat oral ulcers [57].	Aerial parts essential oil from Lahaul-Spiti, Himachal Pradesh, India showed antifungal activity against *Colletotrichum acutatum*, *Colletotrichum fragariae*, and *Colletotrichum gloeosporioides* [53]. Aerial parts essential oil from Uttarakhand, India showed antifungal activity against *Rhizoctonia solani*, *Sclerotium rolfsii*, and *Macrophomina phaseolina* [58]. Aerial parts essential oil from Uttarakhand, India, showed antibacterial activity against *Staphylococcus aureus* (MIC = 6.25 μg/mL) and *Pseudomonas aeruginosa* (MIC = 12.5 μg/mL) [59].	Aerial parts essential oil from Lahaul-Spiti, Himachal Pradesh, India: Aerial parts: camphor (12.6%), artemisia ketone (10.2%), caryophyllene oxide (7.4%), borneol (5.3%) [53]. Aerial parts essential oil from Uttarakhand, India: α-thujone (36.4%), β-thujone (9.4%), germacrene D (6.3%), terpinen-4-ol (6.3%) [58]. Aerial parts essential oil from Garhwal region (Uttarakhand), India (500 m asl): α-thujone (36.9%), β-thujone (8.2%), terpinen-4-ol (7.1%) [60]. Aerial parts essential oil from Garhwal region (Uttarakhand), India (1200 m asl): mequinyl *p*-nitrobenzoate (22.1%), β-eudesmol (12.4%), β-caryophyllene (7.4%) [60]. Aerial parts essential oil from Garhwal region (Uttarakhand), India (2000 m asl): linalool (32.5%), isopulegyl acetate (20.7%), sabinene (6.6%), β-caryophyllene (6.5%) [60]. Leaf oil from Mandi (1044 m asl), Himachal Pradesh, India: caryophyllene oxide (28.6%), methanoazulene (10.9%) [61]. Leaf oil from Manali (2050 m asl), Himachal Pradesh, India: borneol (35.8%), methanozaulene (14.7%), caryophyllene oxide (13.4%) [61]. Leaf oil from Shimla (2210 m asl), Himachal Pradesh, India: camphor (46.9%), β-caryophyllene (13.3%), α-humulene (9.7%) [61].
*Artemisia parviflora* Buch.-Ham. ex D. Don (Asteraceae)	People in Kumaun, Uttarakhand, use the plant to treat skin diseases, burns, cuts, and wounds; fumes are used as insect repellents [19]. The indigenous people of Jammu and Kashmir, India, use the whole plant as a diuretic [62].	None reported for Himalayan essential oils.	Aerial parts essential oil from Kumaun (Uttarakhand), India: germacrene D (41.01%), β-caryophyllene (10.58%), α-humulene (7.86%) [63]. Aerial parts from Pauri, Pauri Garhwal (Uttarakhand), India: β-caryophyllene (15.3%), germacrene D (14.7%), camphor (11.4%), artemisia ketone (7.8%), 1,8-cineole (5.8%) [64].
*Artemisia roxburghiana* Besser (Asteraceae)	In Garhwal Himalaya (Uttarakhand), India, the whole plant extract is used as a tonic and to relieve fever; the plant extract is rubbed on the skin to treat allergic reactions [49]. In the western Himalaya of northern Pakistan, the whole plant extract is used for fever; the powder of whole plant taken for intestinal worms [50].	None reported for Himalayan essential oils.	Aerial parts essential oil from a sample from Kedarnath, Garhwal region, India: β-thujone (65.3%) [45]. Essential oil from aerial parts of plants cultivated in Italy from seeds collected in Kumbu valley, Nepal: 1,8-cineole (16.6%), camphor (15.2%) α-thujone (10.0%) [65]. Aerial parts essential oil from Mussoorie (Uttarakhand), India: borneol (21.2%), linalyl acetate (7.4%), α-humulene (6.7%) [66]. Aerial parts essential oil from Bhatwari (Uttarakhand), India: β-caryophyllene (16.3%), α-thujone (12.0%) [66]. Aerial parts essential oil from Bhaldana (Uttarakhand), India: β-caryophyllene (18.4%), eugenol (16.2%) [66].
*Artemisia scoparia* Waldst. and Kit. (Asteraceae)	The tribal people of the Sewa River area of Jammu and Kashmir, India, use the leaves to treat stomach problems, intestinal worms, indigestion; the leaf powder (mixed with oil) is massaged on joints to releave pain [7]. The Bhots people of Spiti Valley, Himachal Pradesh, India, use a paste made from the leaves to treat earache [49]. Inhabitants of Nanda Devi National Park (Uttarakhand), India apply a paste of the leaves to cuts and wounds [67].	Aerial parts essential oil from Uttarakhand was antibacterial against *Staphylococcus aureus* (MIC = 12.5 μg/mL) and *Bacillus subtilis* (MIC = 12.5 μg/mL) [59].	Aerial parts essential oil from Uttarakhand, India: capillene (42.1%), β-caryophyllene (12.5%), myrcene (9.2%), β-pinene (8.6%), *p*-cymene (6.8%), γ-terpinene (5.3%), 1-phenyl-2,4-pentadiyne (1.1%) [59]. Leaf oil from Milam glacier, Uttarakhand, India: capillene (60.2%), γ-terpinene (11.1%), 1-phenyl-2,4-pentadiyne (1.0%); root oil: capillene (82.9%), 1-phenyl-2,4-pentadiyne (2.6%) [68]. Aerial parts essential oil from Tajikistan: β-pinene (21.3%), 1-phenyl-2,4-pentadiyne (34.2%), myrcene (5.2%), capillene (4.9%) [69].
*Artemisia vulgaris* L. (Asteraceae)	In Nepal, crushed leaves are used to stop nosebleeds; leaves are chewed for mouth ulcers [70]. In the western Himalaya of northern Pakistan, the leaf extract is used for fever [50].	Leaf essential oil not antimicrobial; not cytotoxic [43].	Leaf essential oil from Hetauda Makwanpur, Nepal: α-thujone (30.5%), 1,8-cineole (12.4%), and camphor (10.3%) [43].
*Blumea lacera* (Burm. f.) DC. (Asteraceae)	Inhabitants of the Sewa River area of Jammu and Kashmir, India, use the leaves as an antipyretic, febrifuge, diuretic, and anthelmintic [7].	Essential oil from the aerial parts collected from Baratnagar, eastern Nepal: Cytotoxic (MDA-MB-231, MCF-7, 5637 cells), antimicrobial (*Staphylococcus aureus*, *Candida albicans*, *Aspergillus niger*) [71].	Aerial parts essential oil from Baratnagar, eastern Nepal: (*Z*)-lachnophyllum ester (25.5%), (*Z*)-lachnophyllic acid (17.0%), germacrene D (11.0%), (*E*)-β-farnesene (10.1%) [71].
*Boenninghausenia albiflora* (Hook.) Rchb. ex Meisn. (Rutaceae)	Inhabitants of the Sewa River area of Jammu and Kashmir, India, use the shoots to repel insects; the root used to relieve toothache [7]. People in the Mornaula Reserve Forest of Kumaun (Uttarakhand), India, use roots of the plant to kill fleas, lice, and insects [22].	None reported from Himalayan essential oils.	Aerial parts of essential oils from western Himalaya, India: germacrene D (4.2%–18.2%), τ-cadinol (0.1%–16.3%), β-caryophyllene (4.6%–13.1%), globulol (0.3%–9.2%), β-copaene-4α-ol (0.1%–7.5%), myrcene (2.1%–26.1%) and β-pinene (8.4%–13.8%) [72].
*Callistemon citrinus* (Curtis) Skeels (Myrtaceae) *	The local people in villages of Bhabar tract of Garhwal (Uttarakhand), India, use the plant as an antibacterial and antifungal agent [73].	Essential oil sample from Nepal, Insecticidal (*Drosophila melanogaster*, *Reticulitermes virginicus*) [74]. Essential oil sample from Palampur, Himachal Pradesh was cytotoxic (A549, IC_50_ = 84.0 μg/mL) [75].	Leaf essential oil of plant cultivated in Biratnagar, Nepal: 1,8-cineole (52.1%), α-terpineol (14.7%), eugenol (14.2%) [74]. Leaf essential oil from Nainital, Uttarakhand, India: 1,8-cineole (66.3%), α-pinene (18.7%) [76]. Leaf essential oil of plant cultivated in Palampur, Himachal Pradesh, India: α-pinene (32.3%), limonene (13.1%), α-terpineol (14.6%) [75].
*Cannabis sativa* L. (Cannabinaceae)	Local people in the Rasuwa district of central Nepal [11] and the Tanahun district of western Nepal [12] take a paste made from the plant for stomach problems. In the Humla district of western Nepal, the juice of the leaves and flowers is applied externally on skin diseases, cuts, and wounds; the juice is taken orally to treat diarrhea [44]. In far western Nepal, the local people apply the leaf juice to control bleeding [30]. In the Parvati valley, Himachal Pradesh, India, a leaf paste is used on tumors; leaf powder used on wounds and sores [56].	None reported for Himalayan essential oils.	Leaf essential oil from wild-growing plant in Biratnagar, Nepal: β-caryophyllene (20.4%), α-humulene (7.0%), α-bisabolol (5.8%) [77].
*Carum carvi* L. (Apiaceae)	In far western Nepal, fruits are applied to treat swelling of breast and testicles [30]. In northwestern Nepal, the fruits are chewed to cure stomach problems, fever, swellings, cough, cold, and to kill intestinal worms [44].	None reported for Himalayan essential oils.	Seed oil from Uttarakhand, India: carvone (65.8%–78.8%), limonene (19.4%–31.6%) [78].
*Cassia fistula* L. (Fabaceae)	In the Ayurvedic system, leaves are used as a laxative; applied externally for chilblains, insect bites, swelling, rheumatism, skin eruptions, ringworm, eczema [79].	Leaf oil from Nepal, antifungal (*Aspergillus niger*, MIC = 78 μg/mL; *Candida albicans*, MIC = 313 μg/mL) [80].	Leaf essential oil from Biratnagar, Nepal: eugenol (25.0%), (*E*)-phytol (21.5%), camphor (13.5%), limonene (11.0%), salicyl alcohol (10.4%), linalool (9.9%), and 4-hydroxybenzyl alcohol (8.7%) [80].
*Cassia tora* L. (Fabaceae)	Villagers in the Kali Gandaki watershed area of Nepal use the leaf paste to treat skin disease; to treat stomach ache, the powdered seeds are taken on an empty stomach. [81].	Leaf oil marginally antibacterial (*Bacillus cereus*, *Staphylococcus aureus*, MIC = 625 μg/mL) [82].	Leaf oil from Biratnagar, Nepal: elemol (26.9%), linalool (19.6%), palmitic acid (15.3%) [82].
*Cedrus deodara* (Roxb. ex D. Don) G. Don (Pinaceae)	The people of Baitadi and Darchula districts of far-western Nepal use the wood essential oil externally for scabies [10]. People of the Karnali zone, western Nepal, massage the leaf essential oil to relieve rheumatic pain [83]. In the Sewa River catchment area, Jammu and Kashmir, India, the bark is used as a diuretic, carminative, antiflatulent, and for urinary disorders [7]. People in Kumaun, Uttarakhand, use the fumes from the bark and wood as a snake repellent [19]. People living in the Nanda Devi National Park (Uttarakhand), India, use a decoction of the bark decoction used to treat fever and dysentery [67].	Wood essential oil from Himachal Pradesh, India, insecticidal (*Plutella xylostella* larvae) [84].	Wood essential oil from Himachal Pradesh, India: β-himachalene (38.3%), α-himachalene (17.1%), γ-himachalene (12.6%) [85].
*Centella asiatica* (L.) Urb. (Apiaceae)	The people of Baitadi and Darchula districts of far-western Nepal use the leaf juice to treat urinary problems and cuts and wounds [10]. Tribal people in the Seti River area of western Nepal use the juice from the whole plant to treat fever or urinary tract infections [12]. People in the Jutpani Village, Chitwan district of central Nepal, chew the leaves and stems to relieve headache [13]. People in Kumaun, Uttarakhand, use the leaves used to prepare a brain tonic [19]. Tribal people in the Mornaula Reserve Forest of Kumoun, west Himalaya, India, prepare a tonic made from the whole plant to use as an anthelmintic, to treat dysentery, cholera, diarrhea [22].	None reported for Himalayan essential oils, but an essential oil sample from South Africa has shown antibacterial activity (*Bacillus subtilis*, *Staphylococcus aureus*, *Escherichia coli*, *Pseudomonas aeruginosa*, *Shigella sonnei*) [86].	Aerial parts essential oil from Kathmandu, Nepal: Isocaryophyllene (9.2%–32.3%), β-caryophyllene (7.5%–24.5%), α-humulene (0.1%–17.1%), (*E*)-β-farnesene (1.7%–18.9%) [87].
*Chaerophyllum villosum* Wall. ex DC. (Apiaceae)	In the Lahaul-Spiti district of Himachal Pradesh, the people consume the seeds and leaves to cure stomach pain [88], colds and coughs [89].	Leaf oil from Uttarakhand, antibacterial (*Staphylococcus aureus*, *Streptococcus mutans*), antifungal (*Candida albicans*, *Candida glabrata*) [90]	Leaf oil from Milam glacier (Uttarakhand): γ-terpinene (74.9%), *p*-cymene (10.0%) [91]. Root essential oil from Uttarakhand: carvacrol methyl ether (31.1%), myristicin (19.1%), thymol methyl ether (18.6%), γ-terpinene (11.7%) [92].
*Chenopodium ambrosioides* L. (Amaranthaceae) *	People in the Sudhan Gali area of Pakistan, consider an infusion of herb to be carminative, diaphoretic, and emmenagogue; it is given in cough, pulmonary obstruction, and amenorrhea and is recommended for the expulsion of the dead fetus [93].	None reported for Himalayan essential oils, but an ascaridole-rich essential oil sample from Cuba has shown antiparasitic activity (*Leishmania amazonensis*) [94].	Aerial parts essential oil from Uttarakhand: α-terpinene (8.3-44.7%), *p*-cymene (21.3%–27.1%), ascaridole (17.9%–45.0%) [95].
*Chrysanthemum cinerariifolium* (Trevir.) Vis. (Asteraceae)	The local people in villages of Bhabar tract of Garhwal (Uttarakhand), India, use the plant externally to treat scabies and other skin diseases [73].	None reported for Himalayan essential oils.	Aerial parts: camphor (11.0%), chrysanthenone (7.6%), α-cadinol (4.8%), γ-muurolene (4.6%) and *cis*-chrysanthenol (4.4%) [96].
*Cinnamomum camphora* (L.) J. Presl. (Lauraceae)	The plant is used as an anti-inflammatory, antiseptic, antiviral, bactericidal, counterirritant, diuretic, expectorant, stimulant, rubefacient, vermifuge, decongestant, cough suppressant [97,98].	Essential oil from Pantnagar, Uttarakhand showed antibacterial activity against *Pasturella multocida* [97]. Essential oil from Lucknow, India (dominated by (1*R*)-(+)-camphor), showed antifungal activity against *Choanephora cucurbitarum* [99]. Leaf oil from Nepal, *Artemia salina* lethality (LC_50_ = 2.5 μg/mL), Antifungal (*Aspergillus niger*, MIC = 19.5 μg/mL), Insecticidal (*Chaoborus plumicornis*, *Pieris rapae*, *Drosophila melanogaster*, *Solenopsis invicta* × *richteri*) [100].	Leaf oil from Pantnagar, Uttarakhand: camphor (82.4%) [97]. Leaf oil from Naukuchiatal, Uttarakhand: camphor (81.5%) [101]. Leaf oil from Hetauda, Makwanpur, Nepal: camphor (36.5%), camphene (11.7%), limonene (9.0%), sabinene (6.3%), β-pinene (6.3%) [100].
*Cinnamomum glanduliferum* (Wall.) Meisn. (Lauraceae)	In the Dolakha district, Nepal, a paste of the roots is used to treat wounds and toothache [102]. In northern India, the leaves are used as a stimulant, carminative, and to treat coughs and colds [103].	Leaf oil from northern India, antibacterial: Gram-positive bacteria *Micrococcus luteus* (MIC = 6.86 μg/mL); Gram-negative bacteria, *Escherichia coli* (MIC = 3.40 μg/mL), *Pseudomonas aeruginosa* (MIC = 3.43 μg/mL), and *Aeromonas salmonicida* (MIC = 1.72 μg/mL) [103].	Leaf oil from northern India: 1,8-cineole (41.4%), α-pinene (20.3%), α-terpineol (9.4%), germacrene D-4-ol (6.1%) and α-thujene (5.10%) [103].
*Cinnamomum glaucescens* Hand.-Mazz. (Lauraceae)	In Manipur, India, the powdered bark is used to treat kidney trouble [104].	Fruit oil from Hetauda, Makwanpur, Nepal, nematicidal (*Caenorhabditis elegans*, LC_50_ = 151 μg/mL), insecticidal (*Culex pipiens*, *Reticulitermes virginicus*) [100]. Fruit oil from Lucknow, India, insecticidal (*Callosobruchus chinensis*), antifungal (*Aspergillus flavus*) [105].	Fruit essential oil from Hetauda, Makwanpur, Nepal: methyl (*E*)-cinnamate (40.5%) 1,8-cineole (24.8%), α-terpineol (7.4%) [100]. Commercial fruit essential oil from Nepal: methyl (*E*)-cinnamate (14%) 1,8-cineole (13%), α-terpineol (7%) [106]. Leaf oil from northeast India: elemicin (92.9%) [107].
*Cinnamomum tamala* (Buch.-Ham.) T. Nees and Nees (Lauraceae)	Indigenous people in far western Nepal use the leaves to treat gastric problems [10]. In the Newar community of Kathmandu, Nepal, the leaves are used as a spice and flavorant [42].	Root essential oil from Nepal, insecticidal (*Culex pipiens*, *Solenopsis invicta* × *richteri*) [100]. Leaf oil from Munsyari, Uttarakhand, antibacterial (*Salmonella enterica*, *Escherichia coli*, *Pasturella multocida*); leaf oil from Logaghat, antibacterial (*Pasturella multocida*) [97].	Root essential oil from Hetauda, Makwanpur, Nepal: camphor (35.0%), linalool (10.6%), *p*-cymene (8.5%), *o*-cymene (6.8%), and 1,8-cineole (6.1%) [100]. Leaf oil from Jeolikote, Uttarakhand: (*E*)-cinnamaldehyde (79.4%), (*E*)-cinnamyl acetate (3.7%), linalool (5.4%) [101]. Leaf oil from Munsyari, Uttarakhand: linalool (52.5%), (*E*)-cinnamaldehyde (26.4%), 1,8-cineol (4.2%) [97]. Leaf oil from Lohaghat, Uttarakhand: linalool (29.8%), camphor (44.0%), (*E*)-cinnamaldehyde (14.3%) [97]. Leaf oil from Champawat, Uttarakhand: linalool (24.7%), camphor (25.5%), (*E*)-cinnamaldehyde (30.4%) [97]. Leaf oil from Pannagar, Uttarakhand: eugenol (65.0%) [97]. Leaf essential oil from Uttarakhand: (*E*)-cinnamaldehyde (35.8%–62.3%), (*E*)-cinnamyl acetate (4.7%–22.7%), linalool (5.7%–16.2%) [108].
*Curcuma angustifolia* Roxb. (Zingiberaceae)	In western Nepal, the rhizome paste is applied externally for bruises, pains, injuries, paralysis [30,44]. In eastern Nepal, the Meche people use the dried rhizome powder as an antiseptic in cuts and wounds, and to check bleeding [109].	None reported for Himalayan essential oils.	Rhizome oil from Jagdalpur, central India: xanthorrhizol isomer (12.7%), methyl eugenol (10.5%) [110]. Rhizome oil from Travancore, southern India: camphor (21.3%), germacrone (12.8%) [110].
*Curcuma longa* L. (syn. *Curcuma domestica* Valeton) (Zingiberaceae)	In the Kumaon division of Uttarakhand, the powdered rhizome is used as an antiseptic [19]. In Nepal, the powdered rhizome taken orally to cure gastritis. It is used as a carminative, stimulant, anti-inflammatory, and anthelmintic; externally, the rhizome is mixed with alum and applied as a paste to wounds, inflamed joints and sprains [111,112,113,114].	Leaf oil from Nigeria cytotoxic (Hs578T, PC-3), antimicrobial (*Bacillus cereus*, *Staphylococcus aureus*, *Aspergillus niger*) [115]. Rhizome oil from Nigeria larvicidal (*Anopheles gambiae*) [116].	Rhizome oil from Bhutan: α-turmerone (30%–32%), *ar*-turmerone (17%–26%), β-turmerone (15%–18%). Leaf: α-phellandrene (18.2%), 1,8-cineole (14.6%), *p*-cymene (13.3%) [117]. Rhizome oil from northern India: α-turmerone (44.1%), *ar*-turmerone (5.4%), β-turmerone (18.5%). Leaf: α-phellandrene (53.4%), 1,8-cineole (10.5%), terpinolene (11.5%) [118]. Rhizome oil from Nigeria: α-turmerone (20.8%), *ar*-turmerone (44.4%), β-turmerone (26.5%) [116]. Leaf oil from Nigeria: α-phellandrene (17.5%), 1,8-cineole (4.1%), α-terpinolene (17.8%), *p*-cymene (15.7%), β-pinene (11.7%) [115].
*Cuscuta reflexa* Roxb. (Convolvulaceae)	In far western Nepal, the plant paste used for headache, body ache, itching [10]. In Nepal, the plant is crushed, decocted, and the liquid taken as a treatment for jaundice [12,42,44].	Essential oil from Nepal, antifungal (*Aspergillus niger*) [119].	Essential oil from Nepal: *cis*-3-butyl-4-vinylcyclopentane (26.4%), limonene (5.1%), (*E*)-nerolidol (9.5%) [119].
*Cymbopogon distans* (Nees ex Steud.) Will. Watson (Poaceae)	Aerial parts used in Pakistan (Lakki Marwat) as carminative, prevention of heart diseases, flavoring agent [120].	Aerial parts essential oil from Yunnan, China, insect repellent (*Liposcelis bostrychophila* [booklouse], *Tribolium castaneum* [red flour beetle]) [121].	Aerial parts from from Nainital (Uttarakhand): α-oxobisabolene (68%) [122]. Aerial parts from Munsyari (Uttarakhand): neral/geranial (35.0%), geraniol (9.5%), geranyl acetate (15.0%) [122]. Leaf oil from Thal (Uttarakhand): α-terpinene (24.9%), piperitone (45.3%) [123]. Leaf oil from Jabarkhet (Uttarakhand): limonene (12.6%), bornyl acetate (27.9%) [123]. Leaf oil from Narayan Ashram (Uttarakhand): α-terpinene (22.4%), *cis-p*-menth-2-en-1-ol (22.7%), *trans-p*-menth-2-en-1-ol (10.8%), *cis*-piperitol (13.0%), *trans*-piperitol (5.6%) [123].
*Dodecadenia grandiflora* Nees (Lauraceae)	Ripe fruits are eaten in Garhwal Himalaya (India) [124].	Leaf oil from Uttarakhand, antibacterial (*Staphylococcus aureus*, *Pasteurella multocida*) [125].	Leaf oil from Uttarakhand: germacrene D (26.0%), furanodiene (13.7%) [101].
*Elsholtzia flava* (Benth.) Benth. (Lamiaceae)	In communities of Kathmandu district, Nepal, the leaf juice used to treat insect bites [42]. In the Parvati Valley, the flowers are used to treat skin diseases, diarrhea, and stomachache [126]. The seeds are also used as a curry [127]	None reported for Himalayan plants.	Leaf oil from Uttarakhand: piperitenone (30.8%), carvacrol (4.8%), (*Z*)-anethole (4.4%), γ-elemene (4.8%) [128].
*Eryngium foetidum* L. (Apiaceae) *	Native to the Neotropics; decoction of aerial parts used for pains, fevers, gastrointestinal problems [129].	None reported for Himalayan essential oils.	Aerial parts from far western Nepal: (*E*)-2-dodecenal (58.1%), dodecanal (10.7%), 2,3,6-trimethylbenzaldehyde (7.4%), (*E*)-2-tridecenal (6.7%) [130].
*Eupatorium adenophorum* Spreng. (Asteraceae) *	In Nepal, the leaf juice is used as an antiseptic; to treat cuts and wounds [11,12,42].	Aerial parts essential oil, antibacterial (*Arthrobacter protophormiae*, *Escherichia coli*, *Micrococcus luteus*, *Rhodococcus rhodochrous*, *Staphylococcus aureus*) [25].	Aerial parts: *p*-cymene (16.6%), bornyl acetate (15.6%), amorph-4-en-7-ol (9.6%), camphene (8.9%) [23]. Aerial parts essential oil from northern India: 1-naphthalenol (17.5%), α-bisabolol (9.5%), bornyl acetate (9.0%) [25]. Aerial parts essential oil from northern India: amorph-4-en-7-ol (5.8-17.7%), bornyl acetate (7.6-15.9%), *p*-cymene (0.1-16.6%), 3-acetoxyamorpha-4,7(11)-dien-8-one (0.3-16.3%), α-phellandrene (1.5-9.6%), camphene (0.1-8.9%), α-bisabolol (1.7-7.8%), α-cadinol (0.6-6.2%), and amorph-4,7(11)-dien-8-one (3.2%–5.7%) [131].
*Ficus religiosa* L. (Moraceae)	In far western Nepal, the bark juice is applied for paralysis [30]. In Jammu and Kashmir, India, leaves and young shoots used as a purgative [7].	Leaf oil from Nepal, cytotoxic (MCF-7) [132].	Leaf oil from Kirtipur, Nepal: eugenol (27.0%), itaconic anhydride (15.4%), 3-methyl-cyclopenetane-1,2-dione (10.8%), 2-phenylethyl alcohol (8.0%) [132].
*Filipendula vestita* (Wall. ex G. Don) Maxim. (Rosaceae)	In Kashmir, a leaf paste is applied to wounds [133].	None reported for Himalayan essential oils.	Root essential oil from Milam glacier, Uttarakhand: methyl salicylate (56.0%), salicaldehyde (15.6%), santene (9.4%) [134].
*Gualtheria fragrantissima* Wall. (Ericaceae)	In Newar community of Kathmandu, Nepal, a liquid made from the whole plant is used to treat rheumatism [42].	Leaf oil from Nepal, antibacterial (*Staphylococcus aureus*) [135].	Leaf oil from Godawari forest, Nepal: methyl salicylate (94.2%) [135].
*Hedychium spicatum* Buch.-Ham. Ex Sm. (Zingiberaceae)	In Nepal, a decoction of the rhizome is taken for coughs and colds [42].	Rhizome oil pediculicidal [*Pediculus humanus capitis* (head louse)] [136], antimicrobial (several Gram-positive, Gram-negative bacteria, several fungi) [137].	Rhizome oil from Uttarakhand: 1,8-cineole (15.5%–58.2%), linalool (0.8%–10.6%), terpinen-4-ol (0.7%–15.2%), elemol (0.7%–16.6%), 10-epi-γ-eudesmol (0.2%–13.9%), α-cadinol (4.5%–11.2%) [138]. Leaf oil from Song (Uttarakhand): α-pinene (9.6%), β-pinene (40.9%), 1,8-cineole (11.9%). Root: β-pinene (8.9%), 1,8-cineole (48.7%), α-terpineol (11.8%) [139]. Leaf oil from Bhowali (Uttarakhand): β-pinene (9.3%), 1,8-cineole (34.2%) [139]. Rhizome oil from Song (Uttarakhand): 1,8-cineole (64.0%) [139].
*Inula cappa* (Buch.-Ham. ex D. Don) DC. (Asteraceae)	In Nepal, a decoction of the root is used to treat epilepsy and rheumatism [42].	Aerial parts essential oil from central Himalaya, India, antibacterial (*Enterococcus faecalis*, *Klebsiella pneumoniae*, *Xanthomonas phaseoli*, and *Bacillus subtilis*) [140].	Aerial parts essential oil from central Himalaya, India: β-caryophyllene (27.5%), *cis*-dihydro-mayurone (6.7%), β-bisabolene (6.5%), (*E*)-β-farnesene (5.6%) [140].
*Jasminum mesnyi* Hance (Oleaceae)	In villages of Himachal Pradesh, India, the leaves are used to treat diabetes, central nervous system disorders, gastric disturbance, anorexia, oral sores, nocturnal emission, and for muscular pain [141].	Leaf essential oil from Nepal, *Artemia salina* lethality (LC_50_ = 27.0 μg/mL); not antibacterial; not antifungal [142].	Leaf oil from Nepal: coumarin (48.9%), linalool (14.8%) [142].
*Juglans regia* L. (Juglandaceae)	In Nepal, a decoction of the bark is used for scabies, allergies, toothaches [10], and as an anthelmintic [83]; the nut juice is taken as a tonic [11]. In Uttarakhand a decoction of the bark is used as mouthwash [19]; twigs used as toothbrush for treatment of toothache [67].	Leaf essential oil from Kashmir, antibacterial (*Bacillus subtilis*, *Staphylococcus epidermidis*, *Proteus vulgaris*, *Pseudomonas aeruginosa*, *Staphylococcus aureus*, *Salmonella typhi*, *Escherichia coli*, *Shigella dysenteriae*, *Klebsiella pneumoniae*) [143].	Leaf oil from Kashmir: α-pinene (15.1%), β-pinene (30.5%), β-caryophyllene (15.5%) germacrene D (14.4%), limonene (3.6%) [143]. Leaf oil from Nepal: eugenol (27.5%), methyl salicylate (16.2%), germacrene D (21.4%), (*E*)-β-farnesene (8.2%) [144]. Leaf oil from western Himalaya, type I: β-caryophyllene (47.9%), caryophyllene oxide (8.6%), germacrene D (7.5%) [145]. Leaf oil from western Himalaya, type II: β-pinene (8.5%–39.5%), β-caryophyllene (1.4%–26.9%), germacrene D (5.0%–23.3%), α-pinene (3.1%–18.1%), α-humulene (1.1%–11.8%) [145]. Leaf oil from western Himalaya, type III: germacrene d (16.1%–22.1%), β-caryophyllene (10.4%–13.5%), α-copaene (6.5%–10.1%) [145].
*Juniperus communis* L. (Cupressaceae)	In Himachal Pradesh, people use the twigs to treat joint pains [56]. In Uttarakhand, the leaf paste is applied to skin ailments [67].	None reported for Himalayan essential oils. Berry essential oil (α-pinene-rich) from Portugal, antifungal (*Candida albicans*, *Epidermophyton floccosum*, *Trichophyton rubrum*, *Trichophyton mentagrophytes*, *Microsporum canis*) [146]. Berry essential oil (α-pinene-rich) from Serbia, antibacterial (*Bacillus cereus*) [147]. Berry essential oil (α-pinene-rich) from Croatia, antibacterial (*Bacillus cereus*), antifungal (*Candida albicans*, *Candida kefyr*, *Trichophyton mentagrophytes*, *Trichophyton rubrum*) [148].	Leaf essential oil from Uttarakhand: α-pinene (35.4%), limonene (23.8%) [149]. Berry essential oil from Uttarakhand: α-pinene (10.8%), limonene (15.1%), terpinene-4-ol (8.8%) [149].
*Juniperus indica* Bertol. (Cupressaceae)	In Nepal, the leaves and berries are used to treat fevers, coughs, skin diseases; also used as incense and flavoring [150]. A paste of the leaves and berries applied externally to treat skin diseases [44].	None reported for Himalayan essential oils.	Leaf essential oil from Nepal: sabinene (19.4%–31.3%), terpinen-4-ol (3.7%–13.0%), β-thujone (4.5%–25.8%), *trans*-sabinyl acetate (7.6%–24.3%) [151]. Leaf essential oil from Uttarakhand: sabinene (27.8%), terpinen-4-ol (16.1%), α-pinene (6.3%), γ-terpinene (6.1%) [152]. Berry essential oil from Uttarakhand: sabinene (2.32%), terpinen-4-ol (23.6%), α-pinene (8.8%), γ-terpinene (6.6%) [152].
*Juniperus macropoda* Boiss. (syn. *J. excelsa* M. Bieb.) (Cupressaceae)	In Hamachal Pradesh, the berries used to treat colic, cough, diarrhea, indigestion, skin diseases; the resin is used on ulcers [152]. In Kashmir, the plant is used as incense [153]	Leaf oil from Himachal Pradesh, antifungal (*Candida albicans*, *Colletotrichum acutatum*, *Colletotrichum fragariae*, *Colletotrichum gloeosporioides*), larvicidal (*Aedes aegypti*) [154]. Berry essential oil from Lucknow, larvicidal (*Anopheles stephensi*, *Aedes aegypti*, *Culex quinquefasciatus*) [155].	Leaf essential oil from Chamba, Himachal Pradesh: sabinene (27.5%), terpinen-4-ol (9.4%), cedrol (14.1%) [154]. Leaf essential oil from Hindolkhal, Uttarakhand: β-elemene (42.5%) *trans*-sabinene hydrate (8.8%), α-cubebene (7.9%) [156]. Leaf essential oil from Mussorie, Uttarakhand: α-thujone (22.6%), biformene (7.7%), sabinene (5.8%) [156].
*Juniperus recurva* Buch.-Ham. ex D. Don (Cupressaceae)	Local people in the Rasuwa district of central Nepal use the plant to treat fever, headache, coughs, and colds [11]. In the Humla district of northwestern Nepal, a paste of the leaves and berries is applied externally to treat skin diseases [44]. In the Nubra valley (Jammu and Kashmir), a leaf decoction is used to reduce fever [7].	None reported for Himalayan essential oils.	Leaf oil from eastern Sikkim, India: δ-3-carene (13.6%), δ-cadinene (10.2%), τ-cadinol (5.5%), τ-muurolol (5.5%), α-cadinol (13.1%) [157]. Leaf oil from Langtang National Park, Nepal: sabinene (13.4%), δ-3-carene (23.7%), limonene (18.4%) [157].
*Kyllinga brevifolia* Rottb. (Cyperaceae)	Used medicinally in western Chitwan, Nepal, but use not specified [158]. In the Allai valley, Battagram, Pakistan, the plant is used as fodder [159].	None reported for Himalayan essential oils.	Leaf oil from Biratnagar, Nepal: α-cadinol (40.3%), τ-muurolol (19.5%), germadrene D-4-ol (12.5%) [160].
*Lantana camara* L. (Verbenaceae) *	Native to the Neotropics; decoction taken for rheumatism, diuretic, snakebite, fever, colds; crushed leaves used externally on wounds, ulcers, skin sores [161]. The tribal people of the Sewa River area of Jammu and Kashmir, India, prepare a decoction of the plant to treat tetanus, theumatism, and malaria [7].	Aerial parts essential oil from India, antibacterial (*Arthrobacter protophormiae*, *Micrococcus luteus*, *Rhodococcus rhodochrous*, *Staphylococcus aureus*) [25].	Aerial parts from Uttarahkhand, India: germacrene D (27.9%), germacrene B (16.3%), β-caryophyllene (9.6%) [23]. Aerial parts from India: 3,7,11-trimethyl-1,6,10-dodecatriene (28.9%), β-caryophyllene (12.3%), zingiberene (7.6%), γ-curcumene (7.5%) [25]. Aerial parts from Nepal: davanone (44.4%), (*E*)-nerolidol (13.0%) [162].
*Lawsonia inermis* L. (Lythraceae)	In Nepal, the leaf is used externally for skin wounds and infections [163].	Leaf oil from Nepal not antimicrobial [164].	Leaf oil from Nepal: limonene (20.0%), (*E*)-phytol (27.5%), linalool (7.0%), 1,8-cineole (6.9%) [164]. Leaf oil from Nigeria: α-pinene (18.1%), *p*-cymene (14.7%), 1,8-cineole (58.6%) [165].
*Leucas aspera* (Willd.) Link (Lamiaceae)	In India, a decoction of the plant is taken as an antihelmintic, for headache, asthma, bronchitis; extract taken orally for scabies psoriasis, snake bite; plant used externally as insect repellent [166]; the leaf extract applied to releave toothache [167].	Aerial parts oil from Nepal, antimicrobial (*Bacillus cereus*, *Aspergillus niger*) [168].	Aerial parts essential oil from Nepal: 1-octen-3-ol (30.6%), β-caryophyllene (23.4%), caryophyllene oxide (24.4%) [168].
*Lindera neesiana* (Wall. ex Nees) Kurz (Lauraceae)	In Nepal, the fruits are taken for diarrhea [11]; a paste of the fruit is applied externally to treat boils and scabies [44].	Fruit essential oil from Nepal, antimicrobial (*Staphylococcus aureus*, *Candida albicans*); not cytotoxic [169].	Fruit essential oil from Nepal: geranial (15.1%), neral (11.9%), citronellal (6.7%), 1,8-cineole (8.8%), α-pinene (6.6%), β-pinene (5.6%) [169]. Leaf essential oil from India: methyl chavicol (83.8%), safrole (11.9%) [170]. Branch essential oil: myristicin (70.0%), 1,8-cineole (18.0%) [170].
*Lindera pulcherrima* (Nees) Hook. F. (Lauraceae)	In Newar community of Kathmandu, Nepal, the leaves and branches are used as a spice and flavorant [42].	Leaf essential oil from Uttarakhand, antibacterial (*Staphylococcus aureus*, *Salmonella enterica*) [125].	Leaf essential oil from Utturakhand: curzerenone (17.6%), furanodienone (46.6%) [101].
*Matricaria recutita* L. (syn. *Matricaria chamomilla* L., *Chamomilla recutita* (L.) Rauschert) (Asteraceae) *	Native to southern and eastern Europe; introduced to India during the Mughal period [171]. In France and Germany, it is used to treat digestive ailments (bloating, impaired digestion, eructations, flatulence, gastrointestinal spasms, inflammation); used topically to treat inflammation of skin and mucous membranes, bacterial infections (skin, mouth, gargles); anal and genetal disorders (baths, washes); respiratory irritations (inhalations) [172]. In Nepal, a tea made from flowers used for stomach ailments, as a sleep aid, mild laxative [173].	Aerial parts essential oil, antimicrobial (*Staphylococcus aureus*, *Pseudomonas aeruginosa*, *Candida albicans*) [174].	Aerial parts essential oil from Bara, Nepal: (*E*)-β-farnesene (44.2%), α-bisabolol oxide A (22.3%), (*E*,*E*)-α-farnesene (8.3%) [174]. Floral essential oil from Pantnagar, India: α-bisabolol oxide A (36.5%), α-bisabolol (16.0%), (*E*)-β-farnesene (14.0%), α-bisabolol oxide B (8.6%) [175].
*Mentha arvensis* L. (Lamiaceae)	Local people in the Mornaula Reserve Forest of Kumaun, India use the plant to releave stomach ache, vomiting [22]. In Kashmir, a tea from the leaves is taken to treat gastroenteritis [176]; powder from the aerial parts is taken to treat cough, sore throat, indigestion, and constipation [177]. The Meche people of eastern Nepal chew the leaves to get rid of phlegm from the throat [109]. In Maccheguan, Nepal, the leaves (mixed with *Ocimum sanctum*) are applied externally and taken orally to treat fever, cold, cough [178].	None reported for Himalayan essential oils, but a leaf oil sample from Banaras Hindu University showed broad spectrum antifungal activity against 14 storage fungi, and insecticidal activitity against *Callosobruchus chinensis* [179].	Aerial parts from Kumaon, India: menthol (61.9%–82.2%), menthone (3.6%–19.3%) [180]. Aerial parts of Pantnagar, India: menthol (77.5%–89.3%), menthone (0.3%–7.9%) [181].
*Mentha longifolia* (L.) Huds. (Lamiaceae)	In Jammu and Kashmir, India, the essential oil used for flavorings confectionery [7]; a tea from the leaves is taken as a cooling medicine [182]. In Uttarakhand, India, the herb used for gastrointestinal disorders, cough, colds, and chronic fever [19]. In the Karnali Zone, Nepal, the leaf juice is applied to cuts and wounds as an antiseptic; a leaf decoction is taken to relieve sore throat [83].	None reported for Himalayan essential oils.	Aerial parts essential oil from Kumaon, India: carvone (61.1%–78.7%), dihydrocarveol (0.4%–9.5%), *cis*-carvyl acetate (0.2%–6.4%), germacrene D (1.3%–5.7%) [183]. Leaf essential oil from Sirmaur, Himachal Pradesh, India: piperitenone oxide (54.2%), *trans*-piperitone oxide (24.1%), *cis*-piperitone oxide (7.0%) [184]. Aerial parts essential oil from Tajikistan: *cis*-piperitone oxide (7.8%–77.6%), piperitenone oxide (1.5%–49.1%), carvone (0.0%–21.5%), pulegone (0.3%–5.4%), menthone (0.0%–16.6%) [185].
*Mentha* × *piperita* L. (Lamiaceae)	In Uttarakhand, India, the crushed leaves are used to treat nausea and vomiting [19,186]. Traditional practitioners in Darjeeling, West Bengal, India, use a paste from the whole plant for bodyache [57].	None reported for Himalayan plants, but commercial peppermint oil rich in menthone (27.5%–42.3%) and menthol (18.4%–27.9%) showed antibacterial (*Staphylococcus aureus*, *Listeria monocytogenes*, *Staphylococcus epidermidis*, *Xanthomonas campestris*, *Pseudomonas syringae*) and antifungal (*Candida albicans*) activity [187].	Aerial parts from Kumaon, India: menthol (22.6%–42.8%), menthone (0.8%–33.8%), menthyl acetate (0.6%–32.8%), myrcene (0.0%–15.5%), 1,8-cineole (2.3%–13.9%), menthofuran (0.0%–17.9%) [180].
*Mentha spicata* L. (Lamiaceae)	The people of Baitadi and Darchula districts of far-western Nepal use the plant to treat asthma and urinary complaints [10]. In the Humla district of western Nepal, the plant is chewed for diarrhea and stomachache [44].	None reported for Himalayan essential oils, but leaf oil from Paisalabad, Pakistan showed antibacterial (*Staphylococcus aureus*, *Bacillus cereus*), antifungal (*Aspergillus flavus*, *Rhizopus solani*), and cytotoxic (MCF-7, LNCap) activities [188].	Aerial parts oil from Uttarakhand: carvone (76.7%), limonene (9.6%) [189].
*Morina longifolia* Wall. ex DC. (Caprifoliaceae)	In the Parvati valley, Himachal Pradesh, India, the root powder is applied as a poultice on boils and wounds [56]. In the Chamoli district of Uttarakhand, the people use the fresh leaves to treat boils, cuts and wounds [190]. Indigenous people of Kavrepalanchowk district of central Nepal use the root juice to treat dysentery and diarrhea [191].	Leaf essential oil from Uttarakhand, antibacterial (*Escherichia coli*, *Staphylococcus aureus*, *Proteus vulgaris*, *Klebsiella pneumoniae*, *Bacillus subtilis*, *Pseudomonas aeruginosa*), antifungal (*Alternaria alternata*, *Aspergillus flavus*, *Aspergillus fumigatus*, *Fusarium solani*) [192].	Aerial parts essential oil from Uttarakhand: germacrene D (10.8%), α-pinene (4.8%), bicyclogermacrene (4.3%), α-cadinol (4.3%), (*E*)-citronellyl tiglate (4.2%) β-phellandrene (3.2%) [193]. Aerial parts essential oil from Uttarakhand: β-myrcene (42.5%), bicyclogermacrene (8.9%), germacrene D (6.7%), limonene (6.3%) [194]. Aerial parts essential oil from Uttarakhand: β-myrcene (14.5%–18.7%), geranyl formate (7.5%–10.6%), limonene (5.0%–10.4%), bicyclogermacrene (2.3-%–8.7%) [195].
*Murraya koenigii* (L.) Spreng. (Rutaceae)	In far western Nepal, the leaves used as anthelmintic and in blood disorders [10]. In Uttarakhand, a leaf paste applied to skin diseases [19]. In the Kangra district of Himachal Pradesh, a paste of the branch is applied as a poultice on skin infections [196].	None reported for Himalayan essential oils.	Leaf oil from Dehradun, Uttarakhand: α-pinene (51.7%), sabinene (10.5%), β-pinene (9.8%) [197].
*Nardostachys grandiflora* DC. (Caprifoliaceae)	In far-western Nepal, the rhizome oil is used for headaches; the rhizome is used in epilepsy and mental weakness [10]. In central Nepal, the juice from the whole plant is taken to treat headache and high altitude sickness [11]; the root paste is applied externally to tumors [13]. In northwestern Nepal, a powder or infusion of rhizomes are taken for cough, cold, fever, food poisoning, stomach disorder, intestinal worms, normal headache, and headache from high altitude sickness; a paste is applied externally on wounds; a paste is also used for joint pains and cuts; a root decoction taken early in the morning is beleived to be tonic; the plant is also used as incense [44].	Rhizome oil from Nepal, antimicrobial (*Bacillus cereus*, *Escherichia coli*, *Candida albicans*), cytotoxic (MDF-7) [198].	Rhizome oil from Nepal: β-gurjunene (9.4%), valerena-4,7(11)-diene (7.1%), nardol A (6.0%), 1(10)-aristolen-9β-ol (11.6%), jatamansone (7.9%) [198].
*Neolitsea pallens* (D. Don) Momiy. and H. Hara (Lauraceae)	In the Parbat district of western Nepal, the juice of the fruit is applied externally to treat scabies and exzema [199].	Leaf oil from Uttarakhand not antibacterial [125]	Leaf oil from Uttarakhand: furanogermenone (59.5%), β-caryophyllene (6.6%) [101].
*Nepeta ciliaris* Benth. (Lamiaceae)	Local people in the Kedarnath Wildlife Sanctuary of Uttarakhand use a decoction of the leaves to reduce fever [49].	None reported	None reported
*Nepeta clarkei* Hook. f. (Lamiaceae)	None reported	Aerial parts essential oil from Uttarakhand, antimicrobial (*Pseudomonas aeruginosa*) [200].	Aerial parts essential oil from Malari, Chamoli, Uttarakhand: iridodial β-monoenol acetate (25.3%), β-sesquiphellandrene (22.0%), germacrene D (13.0%), α-guaiene (10.0%) [200]. Aerial parts essential oil from Gulmarg, Kashmir: kaur-16-ene (36.6%), pimara-7,15-dien-3-one (19.7%), caryophyllene oxide (14.1%) [201].
*Nepeta discolor* Royle ex Benth. (Lamiaceae)	In the Bhotiya tribal communities of Niti valley, Uttarakhand, India, a leaf decoction, mixed with honey, is used to treat tuberculosis [190]. In the Nubra valley [38] and the Leh-Ladakh region [202] of Kashmir, a decoction of the leaves is used to treat coughs, colds, and fever.	Essential oil from Uttarakhand, not antimicrobial [200].	Aerial parts essential oil from Malari, Chamoli, Uttarakhand: 1,8-cineole (25.5%), β-caryophyllene (18.6%), *p*-cymene (9.8%) [200].
*Nepeta elliptica* Royle ex Benth. (Lamiaceae)	In Utturakhand, [186] and Jammu and Kashmir [203], an infusion of the seeds is used for digestive disorders.	Aerial parts essential oil from Uttarakhand, antimicrobial (*Pseudomonas aeruginosa*, *Serratia marcescens*, *Candida albicans*, *Trichophyton rubrum*) [200].	Aerial parts essential oil from Clips, Nainital, Uttarakhand: (7*R*)-*trans,trans*-nepetalactone (83.4%) [200]. Aerial parts essential oil from Jammu and Kashmir: β-elemene (23.4%), α-humulene (11.8%), bicyclogermacrene (13.1%) [204].
*Nepeta erecta* (Royle ex Benth.) Benth. (Lamiaceae)	People of the Deosai Plateau of Pakistani Kashmir use the leaves of *N. erecta* to cure cough, cold, fever [205].	Aerial parts essential oil from Uttarakhand, antimicrobial (*Pseudomonas aeruginosa*) [200].	Aerial parts essential oil from Hemkund, Uttarakhand: isoiridomyrmecin (66.7%) [200].
*Nepeta eriostachys* Benth. (Lamiaceae)	People in the Devikund, Bageshwar [206], and Sundardhunga valley [207], Uttrakhand, give an extract of the leaves for fever. The whole plant is used in the Kullu district of Himachal Pradesh for eye complaints [208].	None reported.	None reported.
*Nepeta floccosa* Benth. (Lamiaceae)	People in the cold desert of Ladakh, Kashmir prepare a decoction of the leaves as a remedy for colds, coughs, and fever [202].	None reported.	None reported.
*Nepeta glutinosa* Benth. (Lamiaceae)	In the Nubra valley of Kashmir, a decoction of the leaves is taken to treat diarrhea, pneumonia, and fever [38].	None reported.	None reported.
*Nepeta govaniana* (Wall. ex Benth.) Benth. (Lamiaceae)	In Murari Devi, Himachal Pradesh, a decoction of whole plant taken for colds, influenza, diarrhea, colic, insomnia, mentrual cramps [206]. In Pakistani Kashmir, a decoction of whole plant taken for sore throat, and as a cardiac tonic [207].	Aerial parts essential oil from Uttarakhand, antimicrobial (*Pseudomonas aeruginosa*) [200].	Aerial parts essential oil from Bhundiar, Chamoli, Uttarakhand: isoiridomymecin (35.2%), pregeijerene (20.7%) [200]. Aerial parts essential oil from Uttarakhand: prejeijerene (38%), geijerene (6.8%) [208]. Aerial parts essential oil from Jammu and Kashmir: pregeijerene (56.9%), germacrene D (9.4%), β-caryophyllene (6.1%), torreyol (5.1%) [209].
*Nepeta juncea* Benth. (Lamiaceae)	None reported.	Aerial parts essential oil from Jammu and Kashmir, antifungal (*Aspergillus fumigatus*, *Trichophyton mentagrophytes*, *Trichophyton rubrum*) [210].	Aerial parts essential oil from Jammu and Kashmir: nepetalactone (71.8%) [210].
*Nepeta laevigata* (D. Don) Hand.-Mazz. (Lamiaceae)	In Pakistani Kashmir, an infusion of seeds used to treat dysentery [211]. In the Naran valley, Khyber Pakhtunkhwa, Pakistan, powders of the dried plants used to treat colds, fevers, and headaches [212].	Aerial parts essential oil from Kumaun, Uttarakhand, radical-scavenging (DPPH) [213].	Aerial parts essential oil from Jammu and Kashmir: citronellol (16.5%), β-caryophyllene (10.8%), germacrene D (19.4%), α-bisabolol oxide B (12.4%) [204]. Aerial parts essential oil from Kumaun, Uttarakhand: 1,8-cineole (11.1%), β-caryophyllene (5.7%), caryophyllene oxide (15.2%), manool (7.9%) [214].
*Nepeta leucophylla* Benth. (Lamiaceae)	Local healers in the Baglund district, Nepal, recommend using the root juice for fever [215]. In Utturakhand, a leaf paste used to treat malaria [186].	Aerial parts essential oil from Uttarakhand, antimicrobial (*Pseudomonas aeruginosa*, *Trichophyton rubrum*) [200].	Aerial parts essential oil from Nainital, Uttarakhand: iridodial β-monoenol acetate (25.4%), dihydroiridodial diacetate (18.2%), iridodial dienol diacetate (7.8%) [200].
*Nepeta longibracteata* Benth. (Lamiaceae)	In the Nubra valley of Kashmir, the whole plant is used for stomach disorders [38].	None reported.	None reported.
*Nepeta raphanorhiza* Benth. (Lamiaceae)	None reported.	None reported.	Aerial parts essential oil from Kashmir: (*Z*)-β-farnesene (49.2%), δ-3-carene (12.3%), α-bisabolene (9.4%), germacrene D-4-ol (5.8%) [216].
*Nepeta royleana* R.R. Stewart (Lamiaceae)	None reported.	None reported.	Aerial parts essential oil from Himachal Pradesh: 1,8-cineole (75%) [217].
*Nepeta spicata* Wall. ex Benth. (Lamiaceae)	None reported.	None reported.	Aerial parts essential oil from Uttarakhand: β-caryophyllene (27.0%), linalool (25.1%), germacrene D (20.1%), caryophyllene oxide (10.6%) [218].
*Nyctanthes arbor-tristis* L. (Oleaceae)	In Nepal, a tea made from the leaves is used to reduce fever [42]. In Ayurvedic medicine, the plant is used as an anthelminthic, anti-pyretic, laxative, sedative, and to treat rheumatism and skin ailments [219].	Not antimicrobial [220].	Leaf oil from Nepal: linalool (11.3%), (3Z)-hexenyl benzoate (11.0%), palmitic acid (26.4%), (*E*)-phytol (13.6%) [220]. Bark oil from Nepal: β-eudesmol (17.1%), α-eudesmol (8.7%), palmitic acid (34.3%) [220].
*Ocimum basilicum* L. (Lamiaceae)	Villagers in the Kali Gandaki waternshed area of Nepal use a decoction of seeds to treat urinary disorders; a leaf paste is used externally to treat skin diseases and fungal infections [81].	None reported for Himalayan plants.	Aerial parts essential oil from Nepal: linalool (50.8%–58.3%), geraniol (5.2%–13.7%), eugenol (0.0%–19.1%), τ-cadinol (5.1%–5.9%), 1,8-cineole (0.8%–7.3%) [221].
*Origanum vulgare* L. (Lamiaceae)	The aromatic oil of *O. vulgare* is used as stimulant, rubefacient, and tonic [7]. People in the Parvati valley (Himachal Pradesh), India, apply a paste from the leaves to boils, ulcers, wounds, cuts, burns, and weeping eczema [56]. The plant extract is used by people living in the Nanda Devi National Park (Uttarakhand) to treat bronchitis, coughs, and colds [67]. Local inhabitants of the Kedarnath Wildlife Sanctuary, Uttarakhand, use the leaves to treat toothache and swelling [222]. Local people in the Garhwal Himalaya (Uttarakhand) apply a leaf paste for skin diseases, insect bites, and earache; a leaf decoction is taken for coughs and cold [223]; the powdered leaves are used to treat whooping cough in children [224]. Women in the Gurez Valley of Kashmir take a warm decoction of the plant to alleviate menstrual discomfort [225]. In the Humla district of northwestern Nepal, the dry or fresh plant is boiled with water, liquid is drunk to treat stomachache, diarrhea, dysentry, constipation, toothache, earache and rheumatism. It is also widely used as herbal tea [44].	Thymol-rich essential oil from Uttarakhand, India, antifungal (*Aspergillus flavus* and *Aspergillus niger*) [17]. Thymol-rich essential oil from Uttarakhand, antioxidant and radical scavenging [226].	Aerial parts essential oil from Uttarakhand: thymol (53.2%), *p*-cymene (10.3%), carvacrol (3.9%) [17]. Aerial parts essential oil from Rilkot, Uttarakhand: thymol (82.0%) [227]. Aerial parts essential oil from Kumaon region, Uttarakhand: thymol (40.9%–63.4%), *p*-cymene, (5.1%–25.9%), γ-terpinene (1.4%–20.1%) [228]. Aerial parts essential oil from Milam, Uttarakhand: thymol (68.5%), *p*-cymene (8.5%) [226]. Aerial parts essential oil from Harinagar, Uttarakhand: thymol (41.4%), myrcene (14.2%), α-humulene (9.2%) [226]. Aerial parts essential oil from Bhowali, Uttarakhand: thymol methyl ether (45.2%), thymol (44.6%) [226]. Aerial parts essential oil from Liti, Bageshwar, Uttarakhand: thymol (56.5%), γ-terpinene (20.2%), *p*-cymene (8.7%) [229]. Aerial parts essential oil from Patal Bhuvneshwar, Pithoragarh, Uttarakhand: thymol (45.1%), γ-terpinene (21.8%), linalool (13.1%) [229]. Aerial parts essential oil from Gwaldam, Chamoli, Uttarakhand: thymol (23.3%), (*E*)-β-ocimene (16.0%), *p*-cymene (11.3%), (*Z*)-β-ocimene (8.9%) [229]. Aerial parts essential oil from Uttarakhand: carvacrol (58.3%), γ-terpinene (29.4%), *p*-cymene (8.3%) [229]. Aerial parts essential oil from Aeradev, Almora, Uttarakhand: carvacrol (65.3%), γ-terpinene (15.6%) [229]. Aerial parts essential oil from Kumaon region, Uttarakhand: carvacrol (52.2%–66.1%), γ-terpinene (5.5%–24.1%), *p*-cymene (4.2%–34.4%) [230]. Aerial parts essential oil from Kumaon region, Uttarakhand: carvacrol (53.3%), *p*-cymene (19.2%), γ-terpinene (14.5%) [231]. Aerial parts essential oil from Dhanachuli, Uttarakhand: thymol (29.2%), carvacrol (27.4%), γ-terpinene (10.1%) [227]. Aerial parts essential oil from Dhoulchina, Uttarakhand: thymol (29.7%), carvacrol (20.9%), γ-terpinene (12.4%), *p*-cymene (6.7%) [232]. Aerial parts essential oil from Champawat, Uttarakhand: thymol (35.1%), carvacrol (12.4%), γ-terpinene (14.0%), *p*-cymene (9.8%) [232]. Aerial parts essential oil from Naintal, Uttarakhand: linalool (11.0%), bornyl acetate (7.0%), β-caryophyllene (8.8%), germacrene D (13.3%), germacrene D-4-ol (9.5%) [227]. Aerial parts essential oil from Bhowali, Uttarakhand: linalool (14.7%), α-terpineol (8.4%), bornyl acetate (9.3%), β-caryophyllene (8.7%) [227]. Aerial parts essential oil from Joshimath, Uttarakhand: linalool (34.4%), germacrene D-4-ol (9.6%), α-cubebene (9.4%), β-cubebene (7.8%), terpinen-4-ol (5.7%) [226]. Aerial parts essential oil from Badhangari, Chamoli, Uttarakhand: linalool (28.6%), α-terpineol (20.1%), 1,8-cineole (6.5%) [229]. Aerial parts essential oil from Dronagiri, Almora, Uttarakhand: linalool (29.8%), α-terpineol (11.9%), terpinene-4-ol (10.2%), sabinene (10.0%), γ-terpinene (6.5%), (*E*)-β-ocimene (5.3%) [229]. Aerial parts essential oil from Purara, Bageshwar, Uttarakhand: linalool (34.1%), borneol (12.3%), α-terpineol (9.6%), β-caryophyllene (9.3%), *epi*-α-bisabolol (6.2%), germacrene D (5.5%), selin-11-en-4α-ol (5.1%) [229]. Aerial parts essential oil from Aeradev, Almora, Uttarakhand: (*E*)-β-ocimene (25.4%), linalool (24.2%), (*Z*)-β-ocimene (13.2%), α-terpineol (6.9%), bornyl acetate (6.7%), *epi*-α-bisabolol (6.0%) [229]. Aerial parts: linalool (23.8%), myrcene (18.0%), β-caryophyllene (9.06%), germacrene D (7.4%) [233]. Aerial parts essential oil from Talvari, Chamoli, Uttarakhand: myrcene (26.0%), (*E*)-β-ocimene (15.1%), β-caryophyllene (7.2%), guiaol (7.1%), α-terpineol (7.0%) [229]. Aerial parts essential oil from Gopeshwar, Uttarakhand: terpinen-4-ol (16.8%), linalool (10.1%), β-cubebene (6.7%), germacrene D (5.2%) [226]. Aerial parts essential oil from Badhangari, Chamoli, Uttarakhand: γ-terpinene (43.4%), thymol (17.9%), myrcene (8.8%) [229]. Aerial parts essential oil from Aeradev, Almora, Uttarakhand: γ-terpinene (44.2%), thymol (19.0%) *p*-cymene (12.7%), (*E*)-β-ocimene (8.6%) [229]. Aerial parts essential oil from Kamedi Devi Bageshwar, Uttarakhand: γ-terpinene (40.2%), thymol (39.7%), *p*-cymene (6.1%) [229]. Aerial parts essential oil from Shama, Bageshwar, Uttarakhand: γ-terpinene (45.9%), carvacrol (20.1%), *p*-cymene (14.3%), thymol (5.1%) [229]. Aerial parts essential oil from Purara, Bageshwar, Uttarakhand: sabinene (16.5%), myrcene (14.2%), borneol (13.4%), β-caryophyllene (8.9%), (*E*)-β-ocimene (5.3%) [229]. Aerial parts essential oil from Shama, Bageshwar, Uttarakhand: borneol (15.5%), *epi*-α-bisabolol (12.2%), linalool (12.0%), sabinene (8.1%), bornyl acetate (7.3%), germacrene D (6.7%) [229]. Aerial parts essential oil from Rushi village, Uttarakhand: bornyl acetate (16.8%), germacrene D (11.3%), β-caryophyllene (10.5%), linalool (6.7%) [232]. Aerial parts essential oil from Bhowali, Nainital, Uttarakhand: germacrene D (26.3%), linalool (18.8%), β-caryophyllene (14.6%), *p*-cymene (5.2%) [229]. Aerial parts essential oil from Kilbury, Uttarakhand: β-caryophyllene (13.8%), bornyl acetate (12.6%), linalool (9.7%), germacrene D (6.3%), (Z)-β-ocimene (5.9%) [232].
*Perovskia abrotanoides* Kar. (Lamiaceae)	The plant extract is used by people in the Nubra valley, Jammu and Kashmir, to treat coughs and headache [38].	Essential oil from Karakoram, Jammu and Kashmir, not antifungal [210].	Aerial parts essential oil from Karakoram, Jammu and Kashmir: α-pinene (18.2%–23.2%), 1,8-cineole (24.4%–27.1%), borneol (7.9%–10.4%), β-caryophyllene (5.7%–12.3%), δ-3-carene (4.7%–9.3%) [210].
*Persea duthiei* (King) Kosterm. (Lauraceae)	In India, the tree is not used medicinally; the wood is used for fuel; the leaves are used for fodder; the fruit is edible [234,235].	Leaf oil from Uttarakhand, antibacterial (*Escherichia coli*, *Pasteurella multocida*) [125].	Leaf essential oil from Uttarakhand: α-pinene (10.0%), β-pinene (10.0%), limonene (10.1%), (*E*)-nerolidol (13.2%) [101].
*Persea gamblei* (King ex Hook. f.) Kosterm. (Lauraceae)	In India, this tree is not used medicinally; it is used for firewood [235].	Leaf oil from Uttarakhand, antibacterial (*Staphylococcus aureus*) [125].	Leaf essential oil from Uttarakhand: β-caryophyllene (22.1%), γ-gurjunene (16.8%) [101].
*Persea odoratissima* (Nees) Kosterm. (Lauraceae)	In Nepal, the tree is not used medicinally; the wood is used for fuel; the leaves are used for fodder [236,237].	Leaf oil from Uttarakhand, antibacterial (*Escherichia coli*, *Staphylococcus aureus*, *Salmonella enterica*) [125].	Leaf essential oil from Uttarakhand: α-pinene (16.6%), sabinene (13.1%), β-caryophyllene (10.4%), (*E*)-nerolidol (13.2%) [101].
*Phoebe lanceolata* (Nees) Nees (Lauraceae)	In Uttarakhand, the plant used to treat wounds and sores [238].	Leaf oil from Uttarakhand, antibacterial (*Escherichia coli*) [125].	Leaf essential oil from Uttarakhand: 1,8-cineole (18.2%), β-caryophyllene (27.4%) [101].
*Pinus roxburghii* Sarg. (Pinaceae)	In Kashmir, the bark resin used as expectorant for bronchitis [7]. In far-western Nepal, a paste made from the bark is used to treat burns and scalds; the bark resin is applied to boils [10]. In Uttarakhand, the bark resin is used to treat snake bite and scorpion sting [19].	Cone oil from Nepal: cytotoxic (MCF-7), antifungal (*Aspergillus niger*) [239]	Leaf essential oil from Nepal: β-caryophyllene (31.7%), terpinen-4-ol (30.1%), α-humulene (7.3%) [239]. Bark essential oil from Nepal: β-caryophyllene (34.5%), eugenol (11.4%), linalool (6.4%) [239]. Cone essential oil from Nepal: β-caryophyllene (26.8%), terpinen-4-ol (16.2%), δ-3-carene (6.8%) [239].
*Piper betle* L. (Piperaceae)	In Nepal, the leaves are fried in ghee and taken to treat cough in children [109]. In Himalayan India, the leaves are used to treat headache, sore throat, constipation [73]. In India, betel leaf is used to various conditions, including bad breath, boils, conjunctivitis, constipation, headache, hysteria, itching, mastitis, ringworm, rheumatism, cuts and wounds [240].	Leaf oil from Nepal, cytotoxic (MCF-7) [241].	Leaf essential oil from Nepal: chavibetol (80.5%), chavibetol acetate (11.7%), allylpyrocatechol diacetate (6.2%) [241].
*Pleurospermum angelicoides* (Wall. ex DC.) Benth. ex C.B. Clarke (Apiaceae)	In Uttarakhand, a decoction of the root, mixed with cumin and black pepper, is taken to reduce fever and treat chronic gastric disorders [190,242].	Root, leaf, and floral essential oils from Uttarakhand antifungal (*Candida albicans*); root oil antibacterial (*Salmonella typhi*, *Escherichia coli*, *Streptococcus mutans*); leaf oil antibacterial (*Klebsiella pneumoniae*, *Staphylococcus aureus*, *Streptococcus mutans*, *Bacillus subtilis*); floral oil antibacterial (*Salmonella typhi*, *Klebsiella pneumoniae*, *Streptococcus mutans*) [243].	Root essential oil from Milam Glacier, Uttarakhand: nothoapiole (87.3%) [243]. Leaf essential oil from Milam Glacier, Uttarakhand: limonene (48.4%), α-asarone (23.2%), γ-terpinene (11.0%) [243]. Floral essential oil from Milam Glacier, Uttarakhand: α-pinene (22.3%), α-asarone (20.7%), perilla aldehyde (16.8%), limonene (14.8%) [243].
*Rhododendron anthopogon* D. Don (Ericaceae)	In central Nepal, a tea from dried flowers is taken to treat gastritis and stomach disorders [244]. In the Sunderdhunga valley, Uttarakhand, a decoction of young shoots is given to cure fever [245].	Aerial parts, essential oil from Nepal, antimicrobial (*Bacillus subtilis*, *Mycobacterium tuberculosis*, *Candida pseudotropicalis*); cytotoxic (A-431) [246].	Aerial parts essential oil from Dolakha district, Nepal: α-pinene (37.4%), β-pinene (16.0%), limonene (13.3%), δ-cadinene (9.1%) [246].
*Selinum tenuifolium* Salisb. (Apiaceae)	In the Parvati valley (Himachal Pradesh), India, the smoke produced from the roots is used for killing and repelling insects [56]. People in the Pangi Valley, Himachal Pradesh, use a powder of the roots and umbels to treat swelling and knee pain [247].	None reported for Himalayan essential oils.	Root essential oil from Rhohtang, Himachal Pradesh: Nona-3,5-diyne (85.6%) [248]. Aerial parts essential oil from Chamoli, Uttarakhand: α-bisabolol (71.8%) [249]
*Senecio nudicaulis* Buch.-Ham. ex D. Don (Asteraceae)	In the Almora district of Uttarakhand, the leaf juice is dropped into the eyes to treat conjunctivitis; the leaf paste applied externally to wounds [250].	Aerial parts essential oil from Himachal Pradesh, free-radical-scavenging (DPPH, ABTS) [251].	Aerial parts essential oil from Himachal Pradesh: caryophyllene oxide (25.0%), humulene epoxide-II (21.3%), α-humulene (18.8%), β-caryophyllene (9.7%) [251].
*Senecio rufinervis* DC. (Asteraceae)	In the Tons River valley, Uttarakhesh, a decoction of the leaves is used to relieve stomache ache [252].	None reported for Himalayan essential oils.	Leaf essential oil from Uttarakhand: germacrene D (33.7%), δ-cadinene (5.5%), γ-cadinene (5.5%), germacrene D-4-ol (5.4%) [253]. Root essential oil from Uttarakhand: germacrene D (32.9%), germacrene A (19.5%), δ-elemene (7.6%) [253].
*Skimmia anquetilia* Tayl. and Airy Shaw (Rutaceae)	In far western Nepal, the local people take an infusion of the leaf for headache and freshness [30].	The leaf and floral essential oils from Uttarakhand inhibited egg laying by the beetle, *Caryedon serratus* [254].	Leaf essential oil from Uttarakhand: germacrene B (11.6%), linalool (9.5%), linalyl acetate (7.3%), α-bisabolol (7.2%), β-gurjunene (6.6%) [255]. Floral essential oil from Uttarakhand: β-phellandrene (18.6%), geijerene (15.1%), linalyl acetate (11.2%), linalool (9.4%) [255].
*Skimmia laureola* (DC.) Decne. (Rutaceae)	In Uttarakhand, the leaf used as incense [22]; the leaf paste (with cow urine) is used to treat psoriasis [67]. In the Chail valley of Khyber Pakhtunkhwa, Pakistant, the leaf powder is taken orally with water to treat smallpox, intestinal worms, and colic [256].	Aerial parts essential oil from Jammu and Kashmir, antimicrobial (*Staphylococcus aureus*, *Staphylococcus epidermidis*, *Aspergillus niger*, *Penicillium chrysogenum*) [257]. Leaf oil from Patrak, Pakistan, antispasmodic, antimicrobial (*Micrococcus luteus*, *Streptococcus viridans*, *Pasteurella multocida*; *Tricophyton longifusis*, *Candida albicans*, *Aspergillus flavus*) [258].	Aerial parts essential oil from Jammu and Kashmir: linalyl acetate (33.0%), linalool (25.0%), limonene (8.1%), α-terpineol (5.9%) and geranyl acetate (5.9%) [257]. Aerial parts essential oil from Dalhousie, Himachal Pradesh: linalool (34.9%), linalyl acetate (26.7%), α-terpineol (12.8%), geranyl acetate (6.6%) [154]. Leaf essential oil from Patrak, Pakistan: linalyl acetate (50.5%), linalool (13.1%), geranyl acetate (8.5%), *cis-p*-menth-2-en-1-ol (6.2%) [258].
*Solanum xanthocarpum* Schrad. and J.C. Wendl. (Solanaceae)	The tribal people of the Sewa River area of Jammu and Kashmir, India, use the plant juice to treat dysentery and fever [7].	None reported for Himalayan essential oils.	Fruit essential oil from Kirtipur, Nepal: benzyl benzoate (21.7%), (*E*,*E*)-geranyllinalool (12.6%) [259]. Leaf essential oil from Kirtipur, Nepal: heptacosane (20.0%), (*E*)-phytol (8.4%) [259]. Stem essential oil from Kirtipur, Nepal: palmitic acid (28.9%), heptacosane (12.8%), linoleic acid (10.1%) [259]. Root essential oil from Kirtipur, Nepal: solavetivone (22.9%), palmitic acid (21.0%), linoleic acid (8.2%) [259].
*Stachys sericea* Wall. ex Benth. (Lamiaceae)	In Kashmir, the whole plant taken internally to treat epilepsy [203].	None reported for Himalayan essential oils.	Aerial parts essential oil from Uttarakhand: germacrene D (37.7%), β-caryophyllene (17.4%), δ-cadinene (6.0%) [260].
*Tanacetum gracile* Hook. f. and Thomson (Asteraceae)	In Kashmir, the leaves are used as an anthelmintic (intestinal worms) [62].	Essential oil from Ladakh, Kashmir, cytotoxic, induces apoptosis (HL-60 leukemia, IC_50_ = 27 μg/mL) [261].	Aerial parts essential oil from Ladakh, Kashmir: lavendulol (21.5%), 1,8-cineole (15.2%), (*Z*)-β-ocimene (6.4%) [262]. Aerial parts essential oil from Nalyang valley, Uttarkashi district, Uttarakhand: α-bisabolol (28.0%), chamazulene (8.4%), α-phellandrene (6.9%) [263].
*Tanacetum longifolium* Wall. ex DC. (Asteraceae)	In Kashmir, the root powder is taken with tea to relieve stomach pain [203]. Local inhabitants of the Kedarnath Wildlife Sanctuary, Uttarakhand, use the leaves to treat stomachache and indigestion [222].	Aerial parts essential oil from Milam Glacier, Uttarakhand, antifungal (*Candida albicans*, *Candida glabrata*) [264].	Aerial parts essential oil from Milam Glacier, Uttarakhand: *trans*-sabinyl acetate (43.2%) and *trans*-sabinol (12.7%) [264].
*Tanacetum nubigenum* Wall. ex DC. (Asteraceae)	In Uttarakhand, a decoction of the leaves is used as an antimicrobial [265].	Aerial parts essential oil from Uttarakhand, antibacterial (*Staphylococcus aureus*, *Enterococcus faecalis*), antifungal (*Candida albicans*) [266]. Aerial parts essential oil from Uttarakhand, insecticidal and insect repellent (*Tribolium castaneum*) [267].	Aerial parts from Malari, Chamoli district, Uttarakhand: *cis*-chrysanthenol (37.0%), sabinene (10.7%), *cis*-chrysanthenyl acetate (5.8%), *cis*-chrysanthenyl isobutyrate (5.7%) [268]. Aerial parts essential oil from Milam glacier, Uttarakhand: bornyl acetate (39.7%), borneol (10.6%), (*E*)-β-farnesene (6.6%), 1,8-cineole (5.8%) [269]. Aerial parts essential oil from Pindari glacier, Uttarakhand: linalool oxide acetate (69.4%) [269]. Aerial parts essential oil from Dhol Dhar, Chamoli district, Uttarakhand: 1,8-cineole (30.0%), sabinene (15.6%), eudesmol (11.2%), camphor (8.0%), [263]. Aerial parts essential oil from Gothing, Chamoli district, Uttarakhand: selin-11-en-4α-ol (10.3%), methyl acetopyronone (9.5%), 2,6,8-trimethyl-4-nonanone (8.8%), terpinen-4-ol (7.1%), camphor (6.9%), borneol (5.8%) [267]. Aerial parts essential oil from Burphu, Pithoragarh district, Uttarakhand: borneol (19.8%), 1,8-cineole (10.9%), *cis*-piperitol (10.9%), camphor (9.7%), bornyl acetate (8.1%) [267]. Aerial parts essential oil from Milam glacier, Uttarakhand: bornyl acetate (38.1%), borneol (19.5%), 1,8-cineole (7.3%) [267].
*Thuja orientalis* L. (Cupressaceae)	Women in the Garhwal region of India take a decoction of the bark orally to treat leucorrhea [270]. In Khyber Pakhtunkhwa, Pakistan, the powdered seeds used for tooth ache [271].	Leaf oil from Himachal Pradesh, antifungal (*Alternaria alternata*) [272].	Leaf essential oil from Kangra, Himachal Pradesh: α-pinene (29.2%), δ-3-carene (20.1%), α-cedrol (9.8%), β-caryophyllene (7.5%), α-humulene (5.6%) [272].
*Thymus linearis* Benth. (Lamiaceae)	Tribal people of the Sewa River area of Jammu and Kashmir, India, apply an oil from the herb to the gums for toothache [7]. People in the Mornaula Reserve Forest of Kumaun (Uttarakhand), India, use the whole plant as an anthelmintic and vermicide [22]. In the Humla district of western Nepal, a decoction of the ground aerial parts is drunk to treat cough, cold, stomachache, gastritis, diarrhea, indigestion. It is widely used as herbal tea [44]. The powdered leaf (with honey) is used by people living in the Nanda Devi National Park (Uttarakhand, India), to treat eczema and psoriasis [67].	Essential oils from Pakistani Kashmir, antifungal (*Aspergillus fumigatus*, *Trichophyton mentagrophytes*, *Trichophyton rubrum*) [210]. Essential oil from Gilgit valley, Pakistan, cytotoxic (MCF-7, LNCaP and NIH-3T3) [273].	Aerial parts essential oil from Rupal valley, Pakistani Kashmir: thymol (38.4%), carvacrol (30.7%), γ-terpinene (10.1%) [210]. Aerial parts essential oil from Hunza valley, Pakistani Kashmir: thymol (53.0%), carvacrol (14.4%) [210]. Aerial parts essential oil from Rakaposh, Pakistani Kashmir: geraniol (67.8%), geranyl acetate (16.8%) [210]. Aerial parts essential oil from Gilgit valley, Pakistan: thymol (36.5%), carvacrol (9.5%), thymyl acetate (7.3%), and β-caryophyllene (5.8%) [273]. Aerial parts essential oils from Uttarakhand: thymol (52.3-66.7%), *p*-cymene (1.81-21.6%) and γ-terpinene (1.9-12.5%) [274].
*Thymus serpyllum* L. (Lamiaceae)	Ethnic people of Almora distric of Uttarakhand use the juice of the whole plant orally to treat cough and asthma; the paste of whole plant is used externally to treat arthritis [275].	Aerial parts essential oil from Jammu and Kashmir, antifungal (*Fusarium solani*) [276].	Aerial parts essential oil from Muzaffarabad, Jammu and Kashmir: thymol (16.5-18.8%), 1,8-cineole (14.0%–18.0%) [276]. Aerial parts essential oil from Purara, Uttarakhand: thymol (19.4%–60.1%), γ-terpinene (0.3%–13.8%) and *p*-cymene (3.5%–10.4%) [277]. Aerial parts essential oil from Kattyur valley, Uttarakhand: thymol (58.8%), *p*-cymene (5.7%), thymol methyl ether (4.0%) [278].
*Valeriana hardwickii* Wall. (Caprifoliaceae)	Local people in the Humla district of northwestern Nepal, use an infusion of the root powder for headache, indigestion, diarrhea, dysentery, and for coughs and cold [44]. Ethnic people of Almora distric of Uttarakhand use the plant extract to treat malaria; the leaf paste is used externally to treat boils and eczema [275].	None reported for Himalayan essential oils.	Root/rhizome essential oil from Arunachal Pradesh: bornyl acetate (11.2%), cuparene (7.1%), valeracetate (11.6%), methyl linoleate (21.1%) [279]. Root/rhizome essential oil from Khati village, Uttarakhand: bornyl acetate (20.5%), epoxysesquithujene [280]. Root/rhizome essential oil from Milam, Uttarakhand: valeracetate (17.3%), bornyl acetate (15.3%), methyl linoleate (11.7%), cuparene (10.4%), α-cedrene (6.2%) [281]. Root/rhizome essential oil from Mapang, Pithoragarh, Uttarakhand: bornyl acetate (17.8%), valeracetate (13.3%), 8-epikessyl glycol diacetate (10.6%) [282]. Root/rhizome essential oil from Vishnu Prayag, Chamoli, Uttarakhand: kessanyl acetate (22.2%), maaliol (13.4%), bornyl acetate (7.4%), β-gurjunene (5.4%) [282].
*Valeriana jatamansi* Jones (syn. *Valeriana wallichii* DC.) (Caprifoliaceae)	People in far western Nepal use the root as an anthelmintic and as a tonic [10,283]. Local people in the Rasuwa district of central Nepal Rhizome paste is applied to cuts and wounds and joint problems. Rhizome is chewed to treat sore throat [11]. In the Humla district of western Nepal, Fresh or dry roots are grinded for paste or powder and taken with hot water to treat headache, indigestion, diarrhea and dysentry. It is used in cough and cold. The plant juice or paste is also applied on the body of young babies to protect them from extreme heat-borne diseases [44]. Lay people in the Karnali zone of west Nepal use a decoction of the root to wash wounds [83]. In the villages of Chaubas and Syabru, central Nepal, the rhizome oil is used for rheumatism and dislocation of joints [284]. The local people in the Dolpa district of Nepal use a paste of the rhizome to treat headache, sore throat, and shock; it is also taken as a tonic; leaf and rhizome extracts are applied to boils and burns [285]. Ethnic people of Almora distric of Uttarakhand use the dried root as incense and insecticide [275].	Leaf oil from Kashmir, antifungal (*Microsporum canis*, *Fusarum solani*) [286].	Root/rhizome oil from Uttarakhand: maaliol (64.3%), viridiflorol (7.2%), β-gurjunene (7.2%) [282,287]. Root/rhizome oil from Bageshwar, Uttarakhand: maaliol (53.8%), β-gurjunene (14.2%) [288]. Root/rhizome oil from Uttarkashi, Uttarakhand: maaliol (42.1%), β-gurjunene (20.8%), seychellene (17.6%), α-santalene (8.7%) [288]. Root/rhizome oil from Dehradun, Uttarakhand: maaliol (51.7%), seychellene (13.7%), β-gurjunene (13.2%), α-santalene (6.0%) [288]. Root/rhizome oil from Uttarakhand: patchouli alcohol (40.2%), α-bulnesene (10.7%), seychellene (8.2%), viridiflorol (5.2%) [282,287]. Root/rhizome essential oil from Katarmal forest, Almora, Uttarakhand: patchouli alcohol (36.6%), α-bulnesene (10.0%), seychellene (4.8%) [289]. Root/rhizome oil from Bageshwar, Uttarakhand: patchouli alcohol (63.7%), maaliol (13.3%), seychellene (4.1%), [288]. Root/rhizome oil from Nainital, Uttarakhand: patchouli alcohol (43.1%), seychellene (8.0%), viridiflorol (7.1%), α-bulnesene (6.3%), α-patchoulene (5.7%), maaliol (5.8%) [288]. Root/rhizome oil from Shillong, Meghalaya: patchouli alcohol (57.2%), seychellene (10.8%), α-patchoulene (6.6%), viridiflorol (6.0%), maaliol (5.8%) α-bulnesene (5.2%) [288]. Root/rhizome essential oil from Kosi-Katarmal, Almora, Uttarakhand: patchouli alcohol (52.1%), seychellene (4.5%) [289]. Root/rhizome essential oil from Kullu, Himachal Pradesh: patchouli alcohol (60.2%), azulene (6.7%), seychellene (5.3%) [290]. Root/rhizome essential oil from Mandi, Himachal Pradesh: patchouli alcohol (52.5%), viridiflorol (13.2%) [290]. Root/rhizome essential oil from Mandi, Himachal Pradesh: patchouli alcohol (51.0%), viridiflorol (19.9%) [290]. Root/rhizome essential oil from Chamba, Himachal Pradesh: patchouli alcohol (59.3%), viridiflorol (15.2%) [290]. Root/rhizome essential oil from Cantonment area, Uttarakhand: patchouli alcohol (40.6%), α-bulnesene (12.4%), α-guaiene (9.9%), seychellene (6.2%) [291]. Root/rhizome essential oil from Gwaldam, Uttarakhand: patchouli alcohol (46.2%) [291]. Root/rhizome essential oil from Laubanj, Uttarakhand: patchouli alcohol (60.9%) [291]. Root/rhizome essential oil from Dewalchoura, Uttarakhand: α-bulnesene (23.5%), patchouli alcohol 18.1%), α-guaiene (13.3%), viridiflorol (7.3%) [291]. Root/rhizome essential oil from Shitlakhet, Uttarakhand: patchouli alcohol (28.4%), α-bulnesene (21.4%), α-guaiene (11.2%), seychellene (7.4%) [291]. Root/rhizome essential oil from Jakhera, Uttarakhand: patchouli alcohol (13.4%), α-bulnesene (12.8%), α-guaiene (11.9%), β-gurjunene (7.1%), β-caryophyllene (5.1%) [291]. Root/rhizome essential oil from Kullu, Himachal Pradesh: patchouli alcohol (39.8%), viridiflorol (21.1%), β-gurjunene (6.6%) [290]. Root/rhizome essential oil from Chamba, Himachal Pradesh: patchouli alcohol (27.2%), viridiflorol (27.3%), β-gurjunene (11.7%), α-patchoulene (5.7%) [290]. Root/rhizome essential oil from Chamba, Himachal Pradesh: patchouli alcohol (30.2%), viridiflorol (24.4%), β-gurjunene (13.5%), α-patchoulene (8.2%) [290]. Root/rhizome oil from Almora, Uttarakhand: seychellene (27.4%), maaliol (15.5%), β-gurjunene (13.6%), patchouli alcohol (12.2%), α-santalene (12.0%) [288]. Rhizome essential oil from Bhundiar, Chamoli, Uttarakhand: kanokonyl acetate (42.4%), γ-curcumene (10.7%), *ar*-curcumene (7.2%) [292]. Leaf essential oil from Jammu and Kashmir: maaliol (35.2%), 3-methylvaleric acid (25.7%), β-gurjunene (7.2%) [286]. Leaf essential oil from Uttarakhand: maaliol (39.2%), 3-methylvaleric acid (26.5%) [293].
*Vitex negundo* L. (Verbenaceae)	The tribal people of the Sewa River area of Jammu and Kashmir, India, use the aromatic leaves as a tonic and vermifuge [7]. In far western Nepal, the local people take the leaf juice for stomachache [30]. In the Parvati valley, Himachal Pradesh, India, the people prepare a paste of the leaves with cow urine and apply it to wounds and swellings [56].	None reported for Himalayan essential oils.	Leaf essential oil from Kurukshetra, Haryana, India: ethyl 9-hexadecenoate (28.5%), α-bulnesene (18.0%), caryophyllene oxide (10.2%), β-caryophyllene (5.0%) [294].
*Zanthoxylum armatum* DC. (syn. *Zanthoxylum alatum* Roxb.) (Rutaceae)	The Bhots people of Spiti Valley, Himachal Pradesh, India, use the bark use to relieve toothache [7]. The people of Baitadi and Darchula districts of far-western Nepal use the fruits used to treat colds, coughs, toothaches; the bark is used to stupefy fish [10]. Local people in the Rasuwa district of central Nepal take the pickled fruits for stomach ache and indigestion [11]. In Newar community of Kathmandu, Nepal, the fruit used for antileech, indigestion, spice and flavorant [42].	None reported for Himalayan essential oils.	Fruit essential oil from Pithoragarh, Uttarakhand: linalool (55.3%), limonene (22.5%), methyl cinnamate (8.8%) [295]. Leaf essential from Kumaon, Uttarakhand: 2-undecanone (55.7%), linalool (11.5%), β-caryophyllene (4.6%), 1,8-cineole (4.3%) [296]. Leaf essential oil from Mandi, Himachal Pradesh: linalool (30.6%), 2-decanone (20.9%), 2-tridecanone (8.9%), β-fenchol (9.4%), β-phellandrene (6.0%) [297]. Fruit pericarp oil from Uttar Pradesh: linalool (72%), methyl cinnamate (12.2%), limonene (6.2%), β-phellandrene (5.3%) [298].

* Introduced species
